# Effects of blocking CD24 and CD47 ‘don't eat me’ signals in combination with rituximab in mantle‐cell lymphoma and chronic lymphocytic leukaemia

**DOI:** 10.1111/jcmm.17868

**Published:** 2023-08-31

**Authors:** Andrea Aroldi, Mario Mauri, Daniele Ramazzotti, Matteo Villa, Federica Malighetti, Valentina Crippa, Federica Cocito, Chiara Borella, Elisa Bossi, Carolina Steidl, Chiara Scollo, Claudia Voena, Roberto Chiarle, Luca Mologni, Rocco Piazza, Carlo Gambacorti‐Passerini

**Affiliations:** ^1^ Hematology Division San Gerardo Hospital Monza Italy; ^2^ Department of Medicine and Surgery University of Milano‐Bicocca Monza Italy; ^3^ Lymphoma Unit, Department of Onco‐Hematology IRCCS San Raffaele Scientific Institute Milan Italy; ^4^ Transfusion Medicine Unit San Gerardo Hospital Monza Italy; ^5^ Department of Molecular Biotechnology and Health Sciences University of Torino Torino Italy; ^6^ Department of Pathology Boston Children's Hospital and Harvard Medical School Boston Massachusetts USA; ^7^ Division of Hematopathology European Institute of Oncology (IEO) IRCCS Milan Italy

**Keywords:** chronic lymphocytic leukemia, don't eat me signal, immunotherapy, mantle‐cell lymphoma, non hodgkin lymphoma

## Abstract

Mantle‐cell lymphoma (MCL) is a B‐cell non‐Hodgkin Lymphoma (NHL) with a poor prognosis, at high risk of relapse after conventional treatment. MCL‐associated tumour microenvironment (TME) is characterized by M2‐like tumour‐associated macrophages (TAMs), able to interact with cancer cells, providing tumour survival and resistance to immuno‐chemotherapy. Likewise, monocyte‐derived nurse‐like cells (NLCs) present M2‐like profile and provide proliferation signals to chronic lymphocytic leukaemia (CLL), a B‐cell malignancy sharing with MCL some biological and phenotypic features. Antibodies against TAMs targeted CD47, a ‘don't eat me’ signal (DEMs) able to quench phagocytosis by TAMs within TME, with clinical effectiveness when combined with Rituximab in pretreated NHL. Recently, CD24 was found as valid DEMs in solid cancer. Since CD24 is expressed during B‐cell differentiation, we investigated and identified consistent CD24 in MCL, CLL and primary human samples. Phagocytosis increased when M2‐like macrophages were co‐cultured with cancer cells, particularly in the case of paired DEMs blockade (i.e. anti‐CD24 + anti‐CD47) combined with Rituximab. Similarly, unstimulated CLL patients‐derived NLCs provided increased phagocytosis when DEMs blockade occurred. Since high levels of CD24 were associated with worse survival in both MCL and CLL, anti‐CD24‐induced phagocytosis could be considered for future clinical use, particularly in association with other agents such as Rituximab.

## 1 INTRODUCTION

Mantle‐cell lymphoma (MCL) is a subtype of B‐cell non‐Hodgkin lymphoma (NHL) with heterogeneous behaviour, ranging from indolent phenotype to highly aggressive and drug‐resistant cases with dismal prognosis.^1^ Disease progression and drug resistance may be influenced by tumour microenvironment (TME), since M2‐like immunosuppressive tumour‐associated macrophages (TAMs), which are predominant within TME, are pathologically functional in providing survival signals to MCL cells and TME is known to be involved in disease recurrence also by masking tumoral cells from host immune system.^2^, ^3^, ^4^, ^5^ Even though MCL shows differences in terms of pathogenetic mechanisms and clinical evolution in comparison to chronic lymphocytic leukaemia (CLL), these two B‐cell malignant entities share characteristics regarding B‐cell of origin and the vital interconnection with non‐cancerous cells belonging to TME.^6^ CLL is defined by a high number of circulating abnormal B cells, which is secondary to a balance between increased proliferation and decreased apoptosis activities, sustained by survival signals deriving from TME.^7^ In fact, TME in CLL harbours different cell compounds such as monocyte‐derived nurse‐like cells (NLCs), which resemble the M2‐like macrophagic immunosuppressive profile and turned out to be an important component able to interact with CLL cells, providing an increase of proliferation and survival.^8^

Hence, targeting TAMs within TME has been considered a valid therapeutic strategy in blood cancers.^9^ Cancer‐expressed CD47 was found to be involved in tumour immune escape through interaction with signal regulatory protein‐α (SIRP‐α), expressed by TAMs, being able to quench phagocytosis in a preclinical model of acute leukaemia.^10^ Interestingly, the use of anti‐CD47 monoclonal antibody (mAb), in order to disrupt this checkpoint interaction between cancer cells and TAMs (also known as ‘don't eat me’ signal, DEMs), showed promising clinical activity in pretreated NHL, thanks to the increase in phagocytosis mediated by TAMs.^11^ Other DEMs had been validated in the last years, such as the programmed cell death ligand 1 (PD‐L1) and the major histocompatibility class I complex (MHC‐I).^12^, ^13^ Recently, CD2interle was also demonstrated to be involved in DEMs in solid cancer.^14^

In fact, tumour‐expressed CD24 promoted immune evasion through its interaction with the inhibitory receptor sialic‐acid‐binding Ig‐like lectin 10 (Siglec‐10), expressed by M2‐like TAMs.^14^ In a preclinical model of CD24^+^ solid tumours, the blockade of CD24‐Siglec‐10 interaction with anti‐CD24 mAb showed an increase in TAMs‐associated phagocytosis in vitro and TAMs‐dependent reduction of tumour growth and improvement of survival in vivo.^14^

Furthermore, CD24 can be expressed in some phases of B‐cell differentiation and both MCL and CLL derive from a B‐cell precursor with upregulated CD24.^15^ In this setting, CD24 might play a critical role in the anti‐phagocytic signal, since MCL and CLL represent a subset of B‐cell malignancies with a considerable hostile TME with M2‐like TAMs, able to jeopardize anti‐cancer immunity.^2^, ^3^, ^4^, ^5^, ^6^, ^7^, ^8^

As previously reported, we and others already explored anti‐CD24 activity in MCL, showing an increase in phagocytosis of human MCL in vitro.^16^, ^17^ In this study, we further extended our analysis by considering not only MCL but also CLL, exploring the feasibility of a combination of DEMs blockade with conventional therapies such as anti‐CD20 mAb.

## 2 MATERIALS AND METHODS

### 2.1 Human tumour RNA sequencing analysis

CD24 mRNA expression data for CLL and other subtypes of NHL were obtained from microarray data sets derived from three different studies, available online at Gene Expression Omnibus (GSE22762, GSE132929, GSE6691).^18^, ^19^, ^20^ CD24 raw expression data were normalized per patient based on the mean expression of three housekeeping genes (i.e. ACTB, GAPDH, GUSB) in order to compare CD24 expression from the different studies. CLL data set also provided survival data, which were exploited to assess the impact of CD24 expression on prognosis, whereas another data set was employed to explore this correlation for MCL.^18^, ^21^

### 2.2 Cell culture

MCL cell lines (MINO, Jeko‐1, Granta‐519, JVM‐2, UPN‐1, REC‐1) were provided by Prof. Claudia Voena (University of Torino, Turin, Italy) and Dr. Giovanna Damia (IRCCS Mario Negri, Milan, Italy). CLL cell lines (MEC‐1, PCL‐12) were gifted by Prof. Paolo Ghia (IRCCS San Raffaele, Milan, Italy). Both MCL and CLL cell lines were cultured in RPMI 1640 with 10% fetal bovine serum (FBS), 1 mM L‐glutamine and 100 U/mL penicillin/streptomycin (Euroclone. Milan, Italy), in a humidified, 5% CO_2_ incubator at 37°C. All cell lines were not independently authenticated beyond the identity provided by the Institution of origin and were routinely tested for mycoplasma contamination.

### 2.3 Antibodies and reagents

Unconjugated human anti‐CD24 monoclonal antibody (mAb, clone SN3) was purchased from Novus Biologicals and aliquoted for long‐term storage at −20°C. Unconjugated human anti‐CD47 (clone B6H12.2), anti‐CD20 (clone Rituximab) mAbs and IgG1 isotype control were purchased from BioXCell and stored at +4°C. Unconjugated human anti‐CD45 was purchased from Thermofisher and Human TruStain FcX™ (crystallizable fragment [Fc] Receptor Blocking Solution) was purchased from BioLegend and stored at 4°C. Recombinant human granulocyte‐macrophage colony‐stimulating factor (GM‐CSF), interleukin‐10 (IL‐10), transforming growth factor‐β_1_ (TGF‐β_1_) and interferon‐γ (IFN‐γ) were purchased from PeproTech (Rocky Hill, NJ), whereas lipopolysaccharides reagent (LPS) was purchased from Sigma–Aldrich and stored at −20°C. Further details are listed in Table S1.

### 2.4 Monocyte isolation and macrophage differentiation

Monocytes were purified from Peripheral Blood Mononucleated Cells (PBMCs), collected from healthy volunteers. Briefly, donor blood was diluted with phosphate‐buffered saline (PBS, dilution 1:2) and separated on Lympholyte‐H from Cedarlane in order to get PBMCs. Monocytes were then isolated from PBMCs by CD14 Microbeads isolation kit from Miltenyi Biotec according to the manufacturer's protocol. Isolated monocytes were checked for purity by flow cytometry (CD14+ cells >90% by flow cytometry). Monocytes were then differentiated into macrophages cultured with IMDM (Thermofisher) + 10% AB human serum from Sigma–Aldrich for 7–9 days, at a density of 1.5 × 10^5^ cells/cm^2^, in a humidified 5% CO_2_ incubator at 37°C. At day 0, 50 ng/mL GM‐CSF was added to provide differentiation. Macrophages were stimulated with 50 ng/mL human IL‐10 and 50 ng/mL human TGF‐β_1_ on days 3–4 of differentiation until use on days 7–9, in order to obtain M2‐like phenotype,^14^ whereas 20 ng/mL human IFN‐γ and 50 ng/mL LPS were adopted on days 5–6 to provide M1‐like differentiation.^22^ Unstimulated (M0) macrophages received only GM‐CSF.

### 2.5 Human samples

Primary samples from patients suffering from MCL and CLL were collected at the time of diagnosis with informed consent from San Gerardo Hospital (HSG, Monza, Italy), according to the IRB‐approved protocol (HSG IRB #926, code: CD24.DEM) accepted by Local Ethical Committee. Tumour population was obtained from patients' blood that was diluted with PBS (dilution 1:2) and separated on Lympholyte‐H from Cedarlane to get PBMCs. Tumour cells were then isolated from PBMCs by CD19 Microbeads isolation kit from Miltenyi Biotec according to the manufacturer's protocol. Isolated cancer cells were then checked for purity by flow cytometry (CD45+/CD19+ cells >90% by flow cytometry). Patients‐derived monocytes were selected with the same protocol used for donors' monocytes. Leftover samples from healthy donors for PBMCs and monocytes collection were obtained after plateletpheresis in Transfusion Medicine Unit (HSG, Monza, Italy), after signing informed consent. All the procedures outlined in the study were conducted in accordance with the ethical principles of Helsinki Declaration.

### 2.6 Flow‐cytometry analysis

For analysis of surface antigen expression of MCL/CLL cell lines and primary human samples, the following antibodies were adopted: CD24 (Novus Biologicals), CD47 and CD20 (Miltenyi Biotec). Antibody against CD14 (Miltenyi Biotec) was used to check purity of isolated monocytes, whereas antibodies against CD86, SIRP‐α (Miltenyi Biotec) and Siglec‐10 (Thermofisher) were used to check either M1‐ or M2‐like differentiation and expression of ligands in M0/M1/M2 macrophages of corresponding tumour antigens (SIRP‐α/CD47, Siglec‐10/CD24). Further details are listed in Table S1.

Flow cytometry analysis was performed by harvesting cells of interest and washing twice with fluorescence‐activated cell sorting (FACS) buffer (PBS with 2% FBS). Cells were then stained with antibodies for 20 min in the dark at 4°C. Cells were washed with FACS buffer once and finally stained with 7‐AAD (Miltenyi Biotec) for viability, according to the manufacturer's protocol. Samples were analysed using Attune™ NxT Flow Cytometer (Thermofisher) and data interpreted with FCS Express™ (De Novo Software).

### 2.7 Flow‐cytometry‐based phagocytosis assay

As already reported,^14^ in vitro phagocytosis was based on co‐culture in serum‐free IMDM of target tumoral cells and effector cells (i.e. M2‐like macrophages), at ratio 1:2 (target cells: 100,000; effector cells: 50,000), for 1–2 h, in a humidified 5% CO_2_ incubator at 37°C within ultra‐low‐attachment 96‐well U‐bottom plates (Corning). Briefly, macrophages were collected after detachment with TrypLE Express (Life Technologies) and gentle scraping and finally resuspended with serum‐free IMDM. MCL, CLL cell lines and patients‐derived samples were stained with Carboxyfluorescein Succinimidyl Ester (CFSE. Thermofisher): after washing with PBS, tumoral cells were resuspended at concentration of 20 × 10^6^/mL and labelled with 10 μM of CFSE for 10 min in the dark at 37°C; reaction was then stopped with RPMI 1640 added with 10% FBS for 5 min in the dark at room temperature; cells were then washed twice with PBS and resuspended with serum‐free IMDM.

Phagocytosis assays were performed using anti‐CD24 (clone SN3), anti‐CD47 (clone B6H12.2), anti‐CD20 (clone Rituximab) and IgG_1_ isotype control at a concentration of 10 μg/mL for all the duration of co‐culture analysis (1–2 h). For Fc‐receptor blockade phagocytosis assays, 1.000.000 macrophages were pre‐treated with 5 μL of Human TruStain FcX™ (BioLegend) in 100 μL of staining solution for 45 min at 4°C, and co‐culture conditions were always conducted using FcR blocking solution (5 μL in 100 μL of staining solution for 10^6^ total cells). Co‐culture process was followed by stopping phagocytosis on ice. Cell suspension was washed with ice‐cold PBS once and stained with phycoerythrin (PE)‐labelled anti‐CD11 mAb (clone REA 713; Miltenyi Biotec) for 20 min in the dark at +4°C to label human macrophages. Cells were washed with ice‐cold PBS once and finally stained with 7‐AAD for viability before analysis. Samples were processed using Attune™ NxT Flow Cytometer (Thermofisher) and data were interpreted with FCS Express™ (De Novo Software). Phagocytosis was defined by measuring the number of CD11b^+^/CFSE^+^ macrophages, quantified as the percentage of total CD11b^+^ macrophages. Phagocytosis reaction was performed in technical triplicate and normalized to the highest technical replicate per donor because of heterogenous raw phagocytic activity among different donor‐derived macrophages, as elsewhere explained.^14^

### 2.8 Fluorescent phagocytosis microscopy

Fluorescently CFSE‐labelled MINO cell line was co‐cultured with donor‐derived M2‐like macrophages stained with Hoechst 33342 (Thermofisher), according to the manufacturer's protocol. Cell suspension of 50,000 macrophages and 100,000 MINO cell line were treated with the same antibody concentrations used for flow‐cytometry‐based phagocytosis assays and incubated in a serum‐free IMDM within a 4‐well Nunc™ Lab‐Tek™ II Chamber Slide™ System (Thermofisher) for 1–2 h, in a humidified 5% CO_2_ incubator at 37°C. After incubation, chambers were placed on ice and washed thoroughly with ice‐cold PBS to remove un‐phagocytosed MINO cells. Each chamber was then analysed with an inverted fluorescent microscope (Zeiss AxioObserver; Zeiss) and images were finally processed with ImageJ (NIH). The number of CFSE^+^ cells within macrophages is counted to obtain the phagocytic index, determined as the number of ingested cells per 100 macrophages (counting at least 200 macrophages per condition).

### 2.9 Statistics

The unpaired two‐tailed Mann–Whitney *U* test or Student's *t*‐test was used to measure differences between groups. For multiple group comparisons, one‐way anova or two‐way anova were used to determine statistically significant differences between samples; *p*‐value < 0.05 was considered as a significant threshold and measurements were summarized as mean ± SD (*p < 0.05, **p < 0.01, ***p < 0.001, ****p < 0.0001). Kaplan–Meier analysis with log‐rank (Mantel–Cox) test was employed to estimate the distribution of OS and to compare differences between survival hazards. All analyses were performed with GraphPad Prism 9.

## 3 RESULTS

### 3.1 CD24 expression and correlation with survival

**FIGURE 1:**
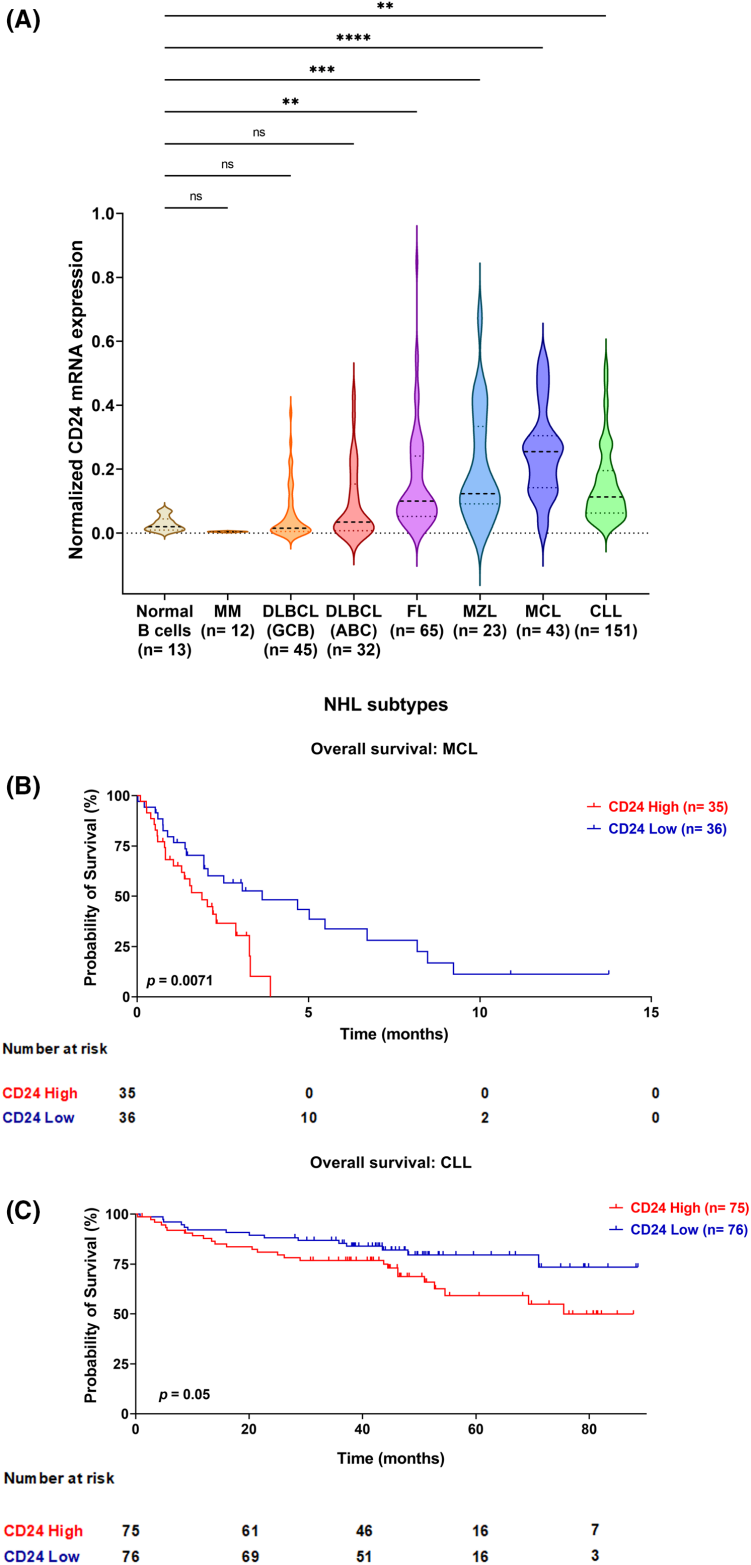
CD24 expression associated with survival curve estimate in MCL and CLL. (A) Violin plot graph showing CD24 RNA expression, normalized to housekeeping genes (i.e. ACTB, GAPDH, GUSB) per patient, in two different cohorts of patients suffering from MCL (*n* = 43) and CLL (*n* = 151), compared to B‐cells from healthy donors (*n* = 12) and other NHL subtypes (Multiple Myeloma [MM, *n* = 12], Diffuse Large B‐Cell Lymphoma [DLBCL] Germinal Centre B‐cell like [CGB, *n* = 45], DLBCL Activated B‐cell‐like [ABC, *n* = 32], Follicular Lymphoma [FL, *n* = 65], Marginal‐zone Lymphoma [MZL, *n* = 23]). CD24 was found higher in MCL and CLL, as well as in FL and MZL, if compared to B cells from healthy subjects. As expected, MM and DLBCL GCB presented low levels of CD24 and DLBCL ABC showed a trend to express higher levels of CD24 (one‐way anova with multiple comparisons correction; CD24 *F*_(7,376)_ = 12.82; ns: not significant, **p < 0.01, ***p < 0.001, ****p < 0.0001). (B and C) Overall survival of patients with MCL (B, *n* = 71) and CLL (C, *n* = 151) with high or low CD24 expression: determination of low and high groups was performed considering gene expression median values. Logrank (Mantel–Cox) test defined a two‐sided *p*‐value. Number of patients at risk in the high‐expression group (red) compared with the low group (blue) is outlined under x axes. High levels of CD24 impacted on survival in MCL and CLL (*p* = 0.0071 and *p* = 0.05, respectively).

CD24 mRNA expression was analysed in a panel of NHL subtypes and CLL from three different microarray data sets.^18^, ^19^, ^20^ CLL and MCL, as well as Follicular Lymphoma (FL) and Marginal‐zone Lymphoma (MZL), showed higher expression of CD24 in comparison to B cells from healthy subjects (Figure 1A). Multiple Myeloma (MM) and Diffuse Large B‐cell Lymphoma (DLBCL) Germinal centre B‐cell‐like (GCB) presented low CD24 levels, whereas DLBCL Activated B‐cell‐like (ABC) showed a trend to express higher levels of CD24(Figure 1A). This pattern was expected since CD24 is expressed during early phases of B‐cell differentiation, with shutdown levels while B cells enter the GC and subsequent upregulation in the next phases, until disappearing when B cells turn into plasma cells.^15^, ^23^ Furthermore, despite the limitations of microarray data sets used, which lacked correlations between CD24 and clinical information, CD24 mRNA expression was found to negatively impact survival in those MCL and CLL patients harbouring higher CD24 levels with respect to patients with lower ones, which might highlight the prognostic relevance of CD24 (Figure 1B,C). Consequently, we first collected a panel of human MCL cell lines in order to assess CD24 surface expression by flow cytometry, showing heterogenous but higher levels of Median Fluorescence Intensity (MFI) if compared to CD24^−^ cancer cell line (Figure S1). CD47 and CD20 MFI were also explored before performing a co‐culture analysis (Figure S1).

### 3.2 Effects of anti‐CD24 alone and combined to anti‐CD47 and anti‐CD20 on phagocytosis and correlation with CD24 antigen density in MCL

Isolation of donor‐derived monocytes was the first step before differentiating macrophages into three different phenotypes, according to the pool of cytokines used (Figure S2A–C). M2‐like macrophages showed the highest levels of Siglec‐10 when compared to M1‐like and M0 macrophages, whereas M1 marker (i.e. CD86) was not detected in this immunosuppressive phenotype (Figure S2B,C). Differences among phenotypes were also confirmed by modifications of morphology, with M1‐like macrophages presenting a star‐like shape in contrast to the small round shape of M0 and M2‐like macrophages (Figure S2D). SIRP‐α was finally checked in our phenotypes before co‐culture assays, showing higher levels in M2‐like than M1‐like phenotype, as already reported (Figure S3A).^24^

Flow‐cytometry‐based phagocytosis assays showed an increase in phagocytosis of CD24^+^ MCF‐7 cell line when treated with anti‐CD24 mAb, depicting a similar rate of phagocytosis if compared to anti‐CD47 mAb; besides, the highest level of phagocytosis was found in case of combination of anti‐CD24 and anti‐CD47 mAbs (Figure S4A,B). Anti‐CD24 mAb did not elicit any off‐target phagocytic activity in CD24^−^ NALM‐6 cell line, showing no differences with the IgG_1_ isotype control group in terms of phagocytosis (Figure S4C).

**FIGURE 2:**
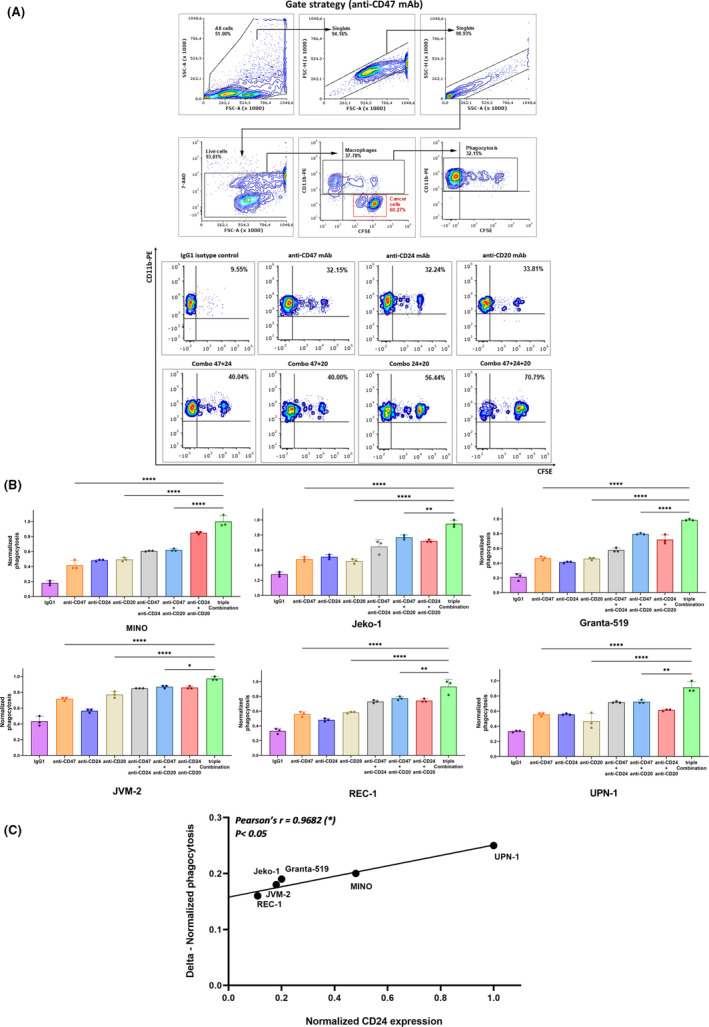
Improvement of phagocytosis of MCL cell lines after simultaneous administration of anti‐CD24/anti‐CD47/antiCD20 mAb and direct correlation between CD24 surface antigen expression and rate of phagocytosis. (A) Upper panel: representative gate strategy performed by ruling out debris and dead cells through morphology and 7‐AAD staining, together with doublet removal. Phagocytosis was counted as a percentage based on the number of 7‐AAD−/CD11b+/CFSE+ events out of all 7‐AAD−/CD11b+ events (total number of macrophages). Depicted flow cytometry plots showed co‐culture assay with donor‐derived M2‐like macrophages and MINO cell line (control condition after administration of anti‐CD47 mAb). Lower panel: representative flow cytometry plot comparison for one MCL cell line (MINO) as regards phagocytosis with IgG1 isotype control, anti‐CD47 mAb, anti‐CD24 mAb, anti‐CD20 mAb (from upper left to upper right side) and combination of anti‐CD24 + anti‐CD47, anti‐CD47 + antiCD20, anti‐CD24 + anti‐CD20, anti‐CD47 + anti‐CD24 + anti‐CD20 mAbs (from lower left to lower right side). (B) Normalized phagocytosis in co‐culture experiment for huMCL. The use of antiCD20 mAb, when combined with anti‐CD24 and anti‐CD47 mAbs (triple Combination), showed the highest phagocytosis rate compared to the other conditions illustrated (one‐way anova with multiple comparisons correction; MINO *F*_7,16_ = 125.5, Jeko‐1 *F*_7,16_ = 69.24, Granta‐519 *F*_7,16_ = 174.1, JVM‐2 *F*_7,16_ = 100.8, REC‐1 *F*_7,16_ = 60.31, UPN‐1 *F*_7,16_ = 42.27; experimental triplicate, *n* = 3 donors; *p < 0.05, **p < 0.01, ****p < 0.0001). (C) Representation of Pearson correlation of delta of normalized phagocytosis (difference between anti‐CD24 and IgG1 isotype control in terms of normalized phagocytosis) that directly correlated with CD24 surface density of MCL. Phagocytosis was much higher for huMCL with higher expression of surface CD24 (normalized phagocytosis obtained by flow cytometry co‐culture assays, using anti‐CD24 mAb (clone SN3); Pearson's *r* = 0.9682 (*) and linear regression *p* < 0.05).

MCL cell lines were then studied to verify an increase in phagocytosis when co‐cultured with donor‐derived M2‐like macrophages and treated with anti‐CD24 alone or in combination with anti‐CD47 and/or anti‐CD20 (Figure 2A,B). The addition of anti‐CD20 mAb (i.e. Rituximab) would help provide further phagocytic stimuli in association with DEMs blockade, taking advantage of Rituximab‐mediated opsonization, as already shown for anti‐CD47 in preclinical and clinical settings.^11^, ^25^ Flow cytometry‐based analysis showed an important increase in phagocytosis when DEMs blockade occurred, as well as a triple combination (anti‐CD47 + anti‐CD24 + anti‐CD20) was employed, representing the highest phagocytic rate obtained (Figure 2B; Figure S5). Anti‐CD24 was demonstrated to upregulate phagocytosis in all conditions and defined a phagocytic activity, working in an antigen‐dependent manner, directly correlated with the CD24 surface density of MCL (Figure 2C).

In order to demonstrate that the increase in phagocytosis after administration of anti‐CD24 mAb was secondary to the blockade of DEMs pathway rather than to Fc‐mediated opsonization, we performed a co‐culture experiment based on the use of M2‐like macrophages previously treated with a cocktail of mAbs able to block Fc‐receptors on macrophage surface (Human TruStain FcX™; BioLegend): this treatment on macrophages would rule out phagocytosis sustained by opsonization process of any antibodies during co‐culture analysis. As expected, in vitro co‐culture conditions showed improved phagocytosis with anti‐CD24 mAb even when macrophages are pretreated with Fc‐receptor blocking solution, depicting similar phagocytic activity shown after use of untreated M2‐like macrophages (Figure S6A,B). Conversely, phagocytosis elicited by anti‐CD20 mAb, whose therapeutic activity is mainly triggered by Fc‐mediated opsonization, is reduced when Fc‐receptor‐blocked macrophages are used (Figure S6A,B).^11^, ^25^ To further validate this hypothesis, we performed co‐culture analysis with another mAb, predominantly harbouring only Fc‐mediated opsonization activity, against CD45, a pan‐expressed antigen by haematopoietic‐derived cells: this antibody provided negligible phagocytosis if compared to anti‐CD24 and anti‐CD47, still suggesting that phagocytosis, after administration of anti‐CD24, is secondary to loss of CD24 signalling rather than to Fc‐mediated opsonization (Figure S6C).

### 3.3 Definition of phagocytic index and validation of anti‐CD24 phagocytosis in Fluorescent microscopy assay

**FIGURE 3:**
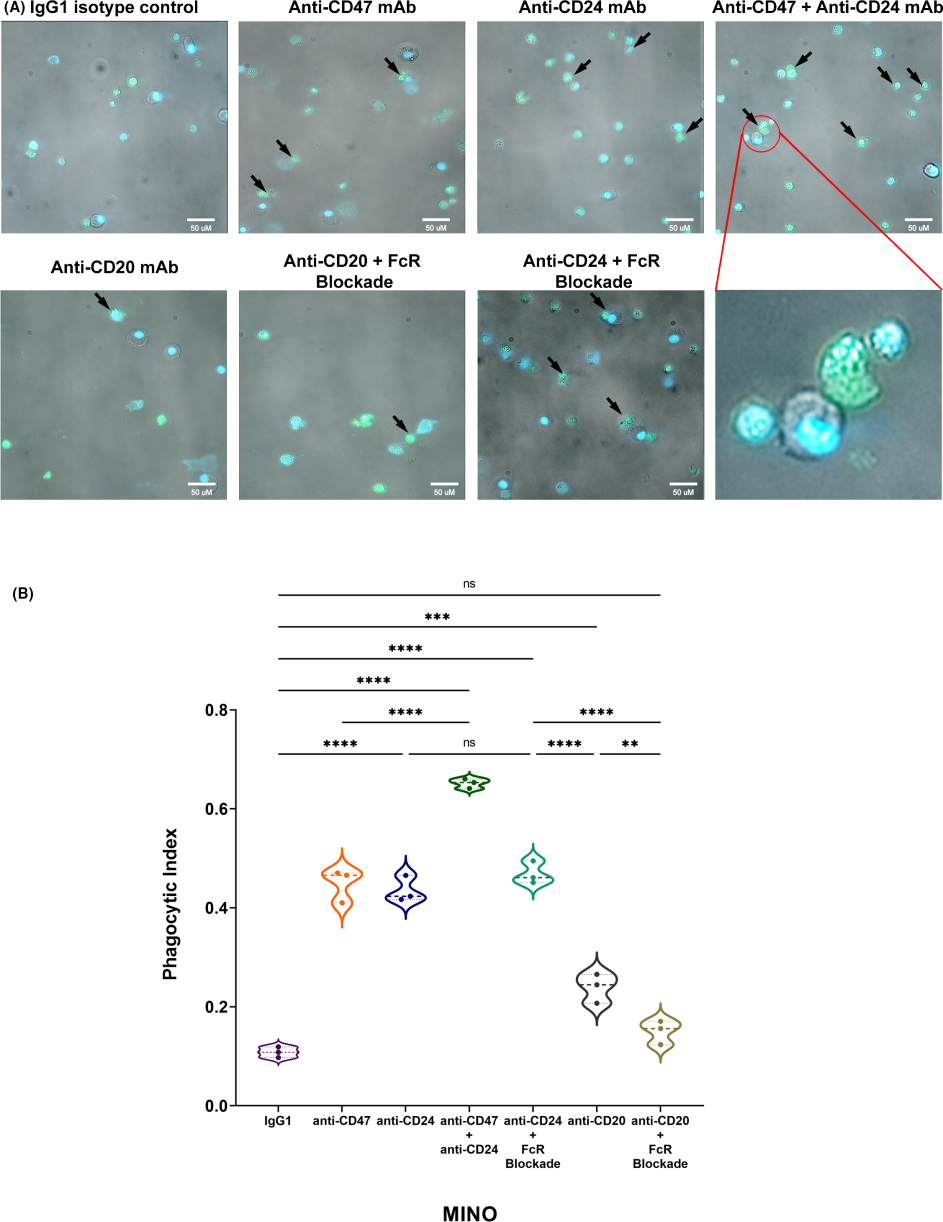
Fluorescent microscopy after incubation of human M2‐like macrophages with MINO and definition of phagocytic index. (A) Representative images of fluorescent microscopy where Hoechst 33342^+^ M2‐like macrophages were incubated with CFSE^+^ MCL cell line (MINO) with different antibodies (IgG1 isotype control, anti‐CD47, anti‐CD24, anti‐CD20, combination of anti‐CD47 + anti‐CD24 mAbs, ± FcR blocking solution), with a higher and similar number of CFSE+ ingested cells in anti‐CD47 and anti‐CD24 groups; higher phagocytosis with the combination of anti‐CD47 and anti‐CD24 mAbs occurred. CFSE+ cells within macrophages (see arrows) in brightfield filter acquisition confirmed macrophage engulfment. Drop of phagocytosis was documented only in anti‐CD20 mAb group when FcR‐blocking solution‐treated macrophages were adopted. (B) Representative violin plot of phagocytic index (number of ingested tumoral cells per 100 macrophages) showing higher and similar levels of phagocytosis in case of occurrence of DEMs blockade, with best improvement of phagocytosis when combined (one‐way anova with multiple comparisons correction; MINO *F*_6,14_ = 202.5; technical triplicate; *n* = 1 donor, one experimental cohort; ns, not significant; **p < 0.01, ***p < 0.001, ****p < 0.0001).

For MCL, in vitro flow cytometry assays have also been implemented with analysis of phagocytosis in fluorescent microscopy, staining MCL cell lines with CFSE and macrophages with Hoechst 33342. The number of CFSE^+^ cells within macrophages was counted to obtain the phagocytic index, determined as the number of ingested cells per 100 macrophages (counting at least 200 macrophages per condition). Even in fluorescent microscopy, phagocytosis was higher when DEMs blockade occurred if compared to IgG_1_ isotype control, thus providing a direct observation of CFSE^+^ cancer cells within macrophages’ cytoplasm after ingestion (Figure 3A,B).

### 3.4 Validation in patient‐derived samples and reproducibility of phagocytosis in CLL

**FIGURE 4:**
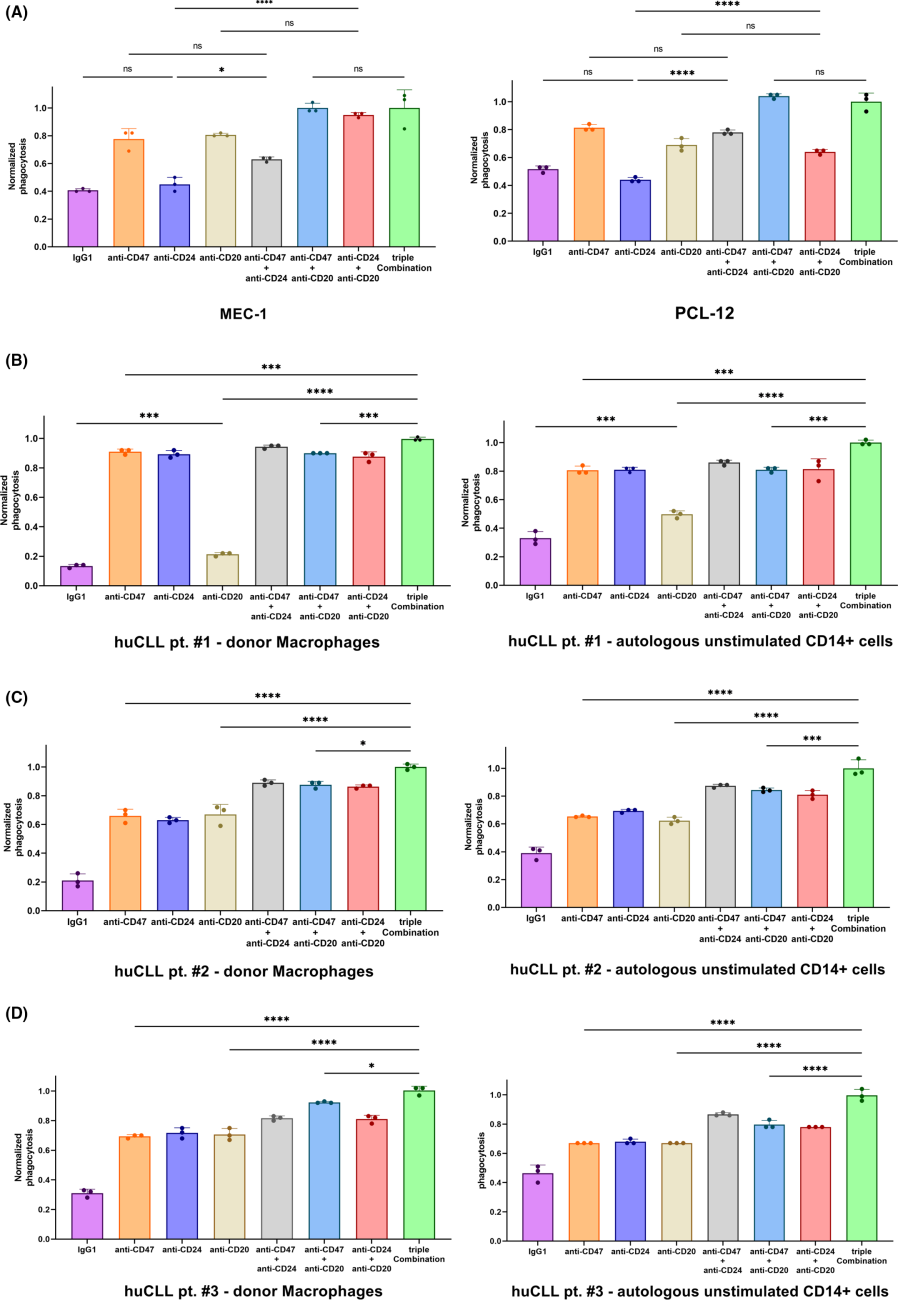
Phagocytosis of CLL patients‐derived samples elicited either by donor‐derived or patients‐derived monocyte–macrophage system (MØ). (A–D) The addition of anti‐CD24 mAb did not provide consistent improvement of phagocytosis for MEC‐1 and PCL‐12, whereas anti‐CD24 increased phagocytosis alone and in case of multiple combinations (anti‐CD47 ± anti‐CD20 mAbs) of CLL patients‐derived samples, even when autologous unstimulated CD14+ cells (monocyte–macrophage system, MØ) were adopted (one‐way anova with multiple comparisons correction; MEC‐1 *F*_7,16_ = 49.12, PCL‐12 *F*_7,16_ = 131.5, huCLL pt. #1 w/donor‐MØ *F*_7,16_ = 1164, huCLL pt. #1 w/auto‐MØ *F*_7,16_ = 110.6, huCLL pt. #2 w/donorMØ *F*_7,16_ = 133.3, huCLL pt. #2 w/auto‐MØ *F*_7,16_ = 110.3, huCLL pt. #3 w/donor‐MØ *F*_7,16_ = 188.3, huCLL pt. #3 w/auto‐MØ *F*_7,16_ = 98.76; technical triplicate, one representative donor; ns, not significant, *p < 0.05, ***p < 0.001, ****p < 0.0001).

We also reproduced functional analysis by co‐culturing donor‐derived macrophages with primary MCL human samples (huMCL pt. #1/pt. #2). We focused our experiment on two patients presenting CD24^low‐density^ and CD24^high‐density^ expression, respectively, confirming in both cases that anti‐CD24 provided upregulation of phagocytosis, particularly when combined with anti‐CD47 and anti‐CD20 (Figures S7 and S8). Since MCL and CLL share the B‐cell of origin, we explored CD24 expression in two CLL cell lines (i.e. MEC‐1, PCL‐12), together with three cases of primary CLL human samples (huCLL). MEC‐1 and PCL‐12 showed heterogeneous expression of CD24, whereas huCLL presented consistent CD24 levels (Figure S9A). CD47 and CD20 expression was additionally checked before co‐culture analysis (Figure S9B). For huCLL samples, we were able to collect enough amount of CD14^+^ cells (attributable to unstimulated monocyte/macrophage system or precursor of NLCs—pre‐NLCs) ready to use as effector cells in co‐culture analysis and redirect these unstimulated autologous effector cells against huCLL cancer cells, since Siglec‐10 levels seemed to be higher if compared to donor‐derived counterparts (Figure S9C). The addition of anti‐CD24 did not provide an increase in phagocytosis when donor‐derived M2‐like macrophages were co‐cultured either with MEC‐1 or PCL‐12 (Figure 4A). Conversely, huCLL samples were sensitive to the addition of anti‐CD24, indicating an increase of phagocytosis in any conditions where anti‐CD24 was added, both with donor‐derived and autologous effector cells (Figure 4B–D; Figure S10). Furthermore, huCLL samples were obtained at the time of diagnosis, but huCLL pt. #1 required prompt therapeutic intervention and received Rituximab for disease control before sample collection: this clinical aspect reflected lower levels of CD20 expression and lower phagocytosis mediated by Rituximab in vitro, while still preserving a combining effect when provided together with anti‐CD24 and anti‐CD47 (Figure 4B; Figures S9B and S10B).

Apparently, in all patient‐derived samples analysed, we did not find any differences in terms of increase of phagocytosis among each combination of double mAbs, raising the question whether the outlined double combination approach had provided higher phagocytosis only through addition of two cumulative Fc‐mediated opsonization mechanisms rather than double DEMs blockade or single DEMs blockade associated with Rituximab‐mediated opsonization action (Figure S7B,C; Figure 4). To address this issue, we performed additional co‐culture analysis considering the condition ‘anti‐CD45 + anti‐CD20 mAbs’ for all patients‐derived samples. As expected, we documented that this condition had a similar phagocytic rate in comparison with ‘anti‐CD20 alone’ and lower levels of phagocytosis if compared to the other double‐mAb conditions. These results would suggest that the increase in phagocytosis, when anti‐CD47 and/or anti‐CD24 were adopted, was due to a different mechanism, like DEMs blockade, rather than combining more antibodies together (Figure S11A–E).

## 4 DISCUSSION

MCL is an aggressive NHL with a dismal prognosis and high probability of relapse after a conventional immunochemotherapeutic regimen.^1^ CLL is instead an incurable chronic B‐cell malignancy with a higher probability of relapse after several lines of therapies and a non‐negligible risk of progression into an aggressive lymphoma.^26^ In these two settings, M2‐like TAMs within TME are known to be involved in cancer cell survival and confer a negative impact on standard treatment efficacy.^2^, ^3^, ^4^, ^5^, ^6^, ^7^, ^8^, ^9^ In recent years, TAMs were shown to be a potential therapeutic target, since DEMs underlined multiple pathogenetic mechanisms explaining tumour progression and disease recurrence.^5^, ^9^

CD47 was the first DEMs antigen identified and it was found upregulated and prognostically relevant in both solid and blood cancers.^9^, ^10^, ^25^ Preclinical models validated the increase of phagocytosis and subsequent tumour growth delay by blocking the CD47/SIRP‐α axis in several blood malignancies.^10^, ^25^, ^27^ These studies subsequently led to the development of trials exploring the safety and efficacy of anti‐CD47 in the clinical setting.^11^, ^28^, ^29^ For instance, anti‐CD47 associated with Rituximab showed promising activity in heavily pre‐treated NHL (DLBCL and Follicular Lymphoma), obtaining clinical response even in those patients with diseases that were refractory to Rituximab.^11^ Notably, anti‐CD47 might restore Rituximab‐induced opsonization, since DEMs could be considered a mechanism of Rituximab resistance and disease recurrence.^11^ Several clinical trials are now ongoing, exploring the safety and efficacy of targeting the CD47/SIRP‐α pathway in various hematologic malignancies, including MCL and CLL.^9^, ^30^, ^31^

In the last decade, other phagocytosis checkpoints have been discovered and CD24 was the last DEMs described in a preclinical model of solid cancer.^12^, ^13^, ^14^ Since CD24 can be expressed during early phases of B‐cell differentiation and both MCL and CLL share similar early B‐cell of origin,^6^, ^15^ blockade of CD24 DEMs could be a promising therapeutic approach to explore in this setting, since TAMs in MCL and NLCs in CLL are strictly interconnected with their corresponding tumoral cells, providing cancer proliferation and immune escape.^2^, ^3^, ^4^, ^5^, ^6^, ^7^, ^8^

We first reported an increase in phagocytosis of MCL cell lines by blocking the CD24/Siglec‐10 axis, suggesting an increase of phagocytosis in an antigen‐dependent manner, as reproduced afterwards by another group.^16^, ^17^ In this current study, we extensively investigated CD24 as DEMs in MCL, showing that anti‐CD24‐induced phagocytosis was mediated by the blockade of the DEMs inhibitory pathway rather than by Fc‐mediated opsonization (Figure S6), as previously shown in solid cancer.^14^ Anti‐CD24 did not show any activity in CD24^−^ cancer cell line (Figure S4C), whereas phagocytosis was restored in co‐culture conditions with all the panels of CD24^+^ MCL cell lines (Figure 2A,B), confirming a direct correlation between the rate of phagocytosis (expressed as delta—normalized phagocytosis, difference between anti‐CD24 and IgG_1_ isotype control in terms of normalized phagocytosis) and CD24 antigen surface density (Figure 2C). The increase in phagocytosis was lower but still considerable in MCL cell lines with CD24^low‐density^ expression (i.e. JVM‐2) as well as in primary human samples (huMCL pt. #1/pt. #2), demonstrating a pivotal combined effect when anti‐CD24 was associated with another DEMs blockade (i.e. anti‐CD47) and B‐specific mAbs (i.e. Rituximab, Figure 2A–C). According to our analysis, we could not assess that the aforementioned multiple combinations induced increase of phagocytosis neither in a synergistic nor in an additive way, but both mAb pairing (i.e. anti‐CD24 + anti‐CD20) and triple mAb matching provided higher responses with respect to single agent conditions and negative control, with the achievement of the highest phagocytosis rate when paired DEMs blockade (i.e. anti‐CD47 + anti‐CD24) was matched with Rituximab (Figure 2A,B). In previous analyses, the combination of mAb against DEMs and other therapeutic agents provided better results than using DEMs blockade alone, considering that targeting phagocytosis checkpoint could improve activity of existing therapies.^9^, ^11^, ^28^ In general, the phagocytosis activity depends on the balance between prophagocytic (e.g. calreticulin, Fc‐mediated opsonization) and anti‐phagocytic surface signals (e.g. CD47, CD24).^28^ Phagocytosis increases when this balance is disrupted in support of a predominance of prophagocytic signals.^28^ This mechanism explained the effects of azacytidine and anti‐CD47 in cases of myelodysplastic syndromes and acute myeloid leukaemia since the hypomethylating agent was shown to upregulate calreticulin levels on cancer cells, thus predominantly boosting prophagocytic signals when DEMs is abated by anti‐CD47.^28^, ^29^ Similarly, in cases of NHL, Rituximab would play as ‘pro‐eat me’ signal, fostering the disruption of the balance in favour of phagocytosis when combined with anti‐CD47, as previously shown.^11^, ^25^

As pertains to CLL, we did not find a benefit from DEMs blockade in MEC‐1 and PCL‐12, expressing inconsistent levels of CD24 if compared to primary human CLL samples, which in turn showed higher CD24 levels and remarkable sensitivity to DEMs targeting (Figure 4; Figure S8). This discrepancy might reflect that MEC‐1 and PCL‐12 represent a prolymphocytic transformation of CLL and EBV‐infected CLL in the progression of disease, respectively. These characteristics could partially explain their phenotypical differences if compared to new‐onset CLL cases.^32^, ^33^ We also demonstrated that huCLL might benefit from the addition of Rituximab to paired DEMs blockade (Figure 4B,C), even in case of low CD20 surface expression, as particularly shown in huCLL pt. #1, previously treated with Rituximab before sample collection (Figure 4B; Figure S9B).

For huCLL, we also found an increase in phagocytosis when pre‐NLCs were employed for co‐culture analysis, showing higher levels of Siglec‐10 than donor‐derived monocytes (Figure 4; Figure S9C). In fact, circulating autologous CD14^+^ cells are the progenitor of resident NLCs and the baseline upregulation of Siglec‐10 might reflect the M2‐like immunosuppressive profile of NLCs within TME, where they interact with CD24^+^ cancer cells, receiving anti‐phagocytic stimuli.^7^, ^8^ Disruption of this axis might therefore restore NLCs‐mediated phagocytosis, leading to the clearance of cancer cells. Nevertheless, further studies are needed to confirm our preliminary data of Siglec‐10 upregulation in a large‐scale analysis of human samples.

Finally, as shown in Figure 1, we found in large microarray data sets that CD24 was upregulated in MCL and CLL patients, with respect to B cells from healthy donors and upregulation of CD24 displayed a potential prognostic value, being associated with poor outcome (Figure 1B). Nevertheless, the correlation between clinicopathological features and CD24 expression warrants further future studies in these NHL subtypes.

Furthermore, the increase in phagocytosis we demonstrated in vitro in the case of CD24.DEMs blockade, particularly in combination with biological agents such as Rituximab, requires confirmation with in vivo models before undertaking use in the clinical setting. In fact, the clinical use of antibodies directed against CD24 would have a significant role in managing relapsed and refractory patients with MCL and CLL, where therapeutic options have been exhausted and the prognosis is inevitably poor.^1^, ^26^

## AUTHOR CONTRIBUTIONS

**Andrea Aroldi:** Conceptualization (lead); data curation (lead); formal analysis (lead); funding acquisition (supporting); investigation (lead); methodology (lead); project administration (lead); resources (lead); software (lead); supervision (lead); validation (lead); visualization (lead); writing – original draft (lead); writing – review and editing (lead). **Mario Mauri:** Data curation (supporting); formal analysis (supporting); methodology (supporting). **Daniele Ramazzotti:** Data curation (supporting); formal analysis (supporting); methodology (supporting). **Matteo Villa:** Data curation (supporting); formal analysis (supporting); methodology (supporting). **Federica Malighetti:** Data curation (supporting); formal analysis (supporting); methodology (supporting). **Valentina Crippa:** Data curation (supporting); formal analysis (supporting); methodology (supporting). **Federica Cocito:** Data curation (supporting); formal analysis (supporting); methodology (supporting). **Chiara Borella:** Data curation (supporting); formal analysis (supporting); methodology (supporting). **Elisa Bossi:** Data curation (supporting); formal analysis (supporting); methodology (supporting). **Carolina Steidl:** Data curation (supporting); formal analysis (supporting); methodology (supporting). **Chiara Scollo:** Data curation (supporting); formal analysis (supporting); methodology (supporting). **Claudia Voena:** Investigation (supporting). **Roberto Chiarle:** Investigation (supporting); methodology (supporting); project administration (supporting). **Luca Mologni:** Data curation (supporting); formal analysis (supporting); funding acquisition (supporting); investigation (supporting); methodology (supporting). **Rocco Piazza:** Data curation (supporting); formal analysis (supporting); funding acquisition (lead); investigation (supporting); methodology (supporting). **Carlo Gambacorti‐Passerini:** Data curation (supporting); formal analysis (supporting); funding acquisition (lead); investigation (supporting); methodology (supporting).

## FUNDING INFORMATION

This study was supported by the following fundings: ‘Associazione Italiana Ricerca sul Cancro (IG‐20112 to CG‐P)’ and ‘Associazione Italiana Ricerca sul Cancro (IG‐22082 to RP)’. The authors independently developed, directed and are fully responsible for all content of this manuscript.

## CONFLICT OF INTEREST STATEMENT

The authors declare no conflicts of interest.

## Supporting information


Data S1.

## Data Availability

The data that support the findings of this study are available from the corresponding author upon reasonable request.
