# Supplementary material for: Effects of blocking CD24 and CD47 ‘don't eat me’ signals in combination with rituximab in mantle‐cell lymphoma and chronic lymphocytic leukaemia
Source: J Cell Mol Med. 2023 Aug 31;27(20):3053–64. doi: 10.1111/jcmm.17868 (PMC10568669; doi:10.1111/jcmm.17868)
Supplement: Supplementary file 1 — Data S1. [file JCMM-27-3053-s001.docx]

Supplemental information to Aroldi et al:

# Effects of blocking CD24 and CD47 “don’t eat me” signals in combination with Rituximab in mantle-cell lymphoma and chronic lymphocytic leukemia

Andrea Aroldi, Mario Mauri, Daniele Ramazzotti, Matteo Villa, Federica Malighetti, Valentina Crippa, Federica Cocito, Chiara Borella, Elisa Bossi, Carolina Steidl, Chiara Scollo, Claudia Voena, Roberto Chiarle, Luca Mologni, Rocco Piazza and Carlo Gambacorti-Passerini

# Supplemental Table

**Supplemental Table 1.** Antibodies used with corresponding clone, purpose and company (Flow Cytometry, FC).

| **Antibody** | **Clone** | **Purpose** | **Company** |
| --- | --- | --- | --- |
| **Anti-human CD24** | SN3  (conjugated) | FC | Novus  Biologicals |
|  | SN3  (unconjugated) | Treatment | Novus  Biologicals |
| **Anti-human CD47** | REA220  (conjugated) | FC | Miltenyi Biotec |
|  | B6H12.2  (unconjugated) | Treatment | BioXCell |
| **Anti-human CD20** | REA780  (conjugated) | FC | Miltenyi Biotec |
|  | Rituximab  (unconjugated) | Treatment | BioXCell |
| **Anti-human CD45** | 5B1  (conjugated) | FC | Miltenyi Biotec |
|  | HI30  (unconjugated) | Treatment | Thermofisher |
| **Human IgG_1_ isotype**  **control** | N/A  (Cat. # BE0297) | Treatment | BioXCell |
| **Human TruStain**  **FcX™** | N/A  (Cat. # 422302) | Treatment | BioLegend |
| **Anti-human CD11b** | REA713  (conjugated) | FC | Miltenyi Biotec |
| **Anti-human CD14** | REA599  (conjugated) | FC | Miltenyi Biotec |
| **Anti-human Siglec-10** | 5G6  (conjugated) | FC | Thermofisher |
| **Anti-human SIRP-α** | REA144  (conjugated) | FC | Miltenyi Biotec |
| **Anti-human CD86** | FM95  (conjugated) | FC | Miltenyi Biotec |

# Supplemental Figures

**AA**

**B CD24 surface expression**


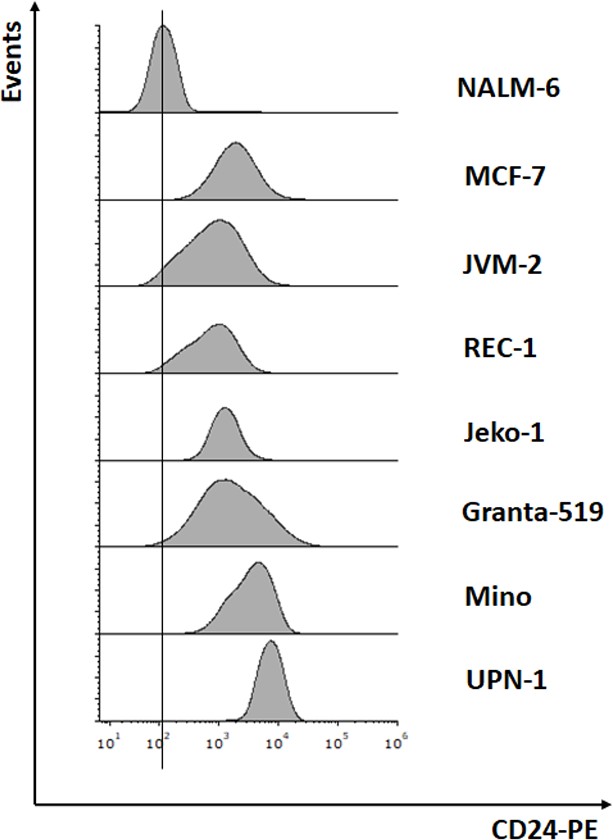


**C**

| **Comparison to NALM-6 MFI** | **Adjusted p Value** | **Summary** |
| --- | --- | --- |
| MCF-7 | <0.0001 | **** |
| JVM-2 | <0.0001 | **** |
| REC-1 | <0.0001 | **** |
| Jeko-1 | <0.0001 | **** |
| Granta-519 | <0.0001 | **** |
| MINO | <0.0001 | **** |
| UPN-1 | <0.0001 | **** |

**D**

1. **CD47 surface expression**
2. **CD20 surface expression**


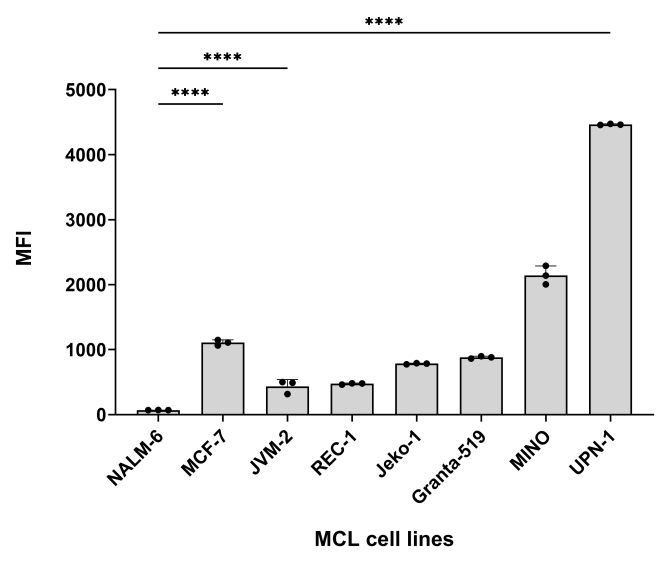


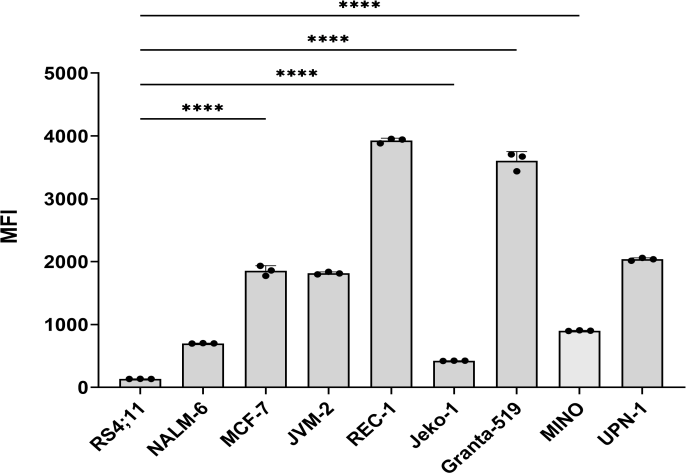

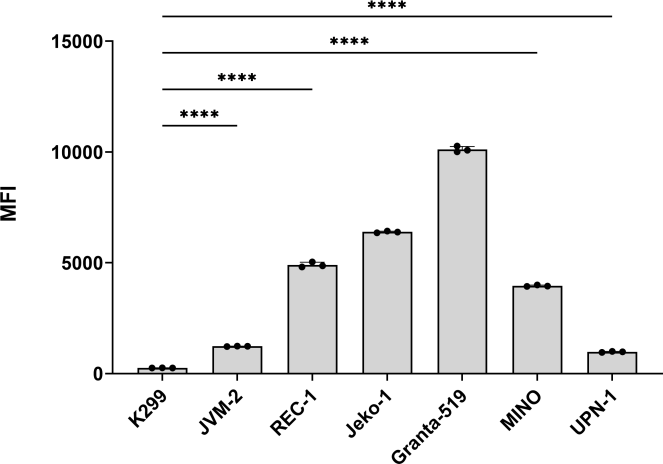


**Supplemental Figure 1**

| **Comparison to RS4;11 MFI** | **Adjusted p Value** | **Summary** |
| --- | --- | --- |
| NALM-6 | <0.0001 | **** |
| MCF-7 | <0.0001 | **** |
| JVM-2 | <0.0001 | **** |
| REC-1 | <0.0001 | **** |
| Jeko-1 | <0.0001 | **** |
| Granta-519 | <0.0001 | **** |
| MINO | <0.0001 | **** |
| UPN-1 | <0.0001 | **** |

| **Comparison to K299 MFI** | **Adjusted p Value** | **Summary** |
| --- | --- | --- |
| JVM-2 | <0.0001 | **** |
| REC-1 | <0.0001 | **** |
| Jeko-1 | <0.0001 | **** |
| Granta-519 | <0.0001 | **** |
| MINO | <0.0001 | **** |
| UPN-1 | <0.0001 | **** |

# Supplemental Figure 1

**Surface expression of CD24, CD47 and CD20 on human Mantle-cell lymphoma cell lines. A-B)** Representative histogram (A) and Median Fluorescence Intensity (MFI, B), by flow cytometry, of CD24 expression in a panel of MCL cell lines (MCL: JVM-2, REC-1, Jeko-1, Granta-519, MINO, UPN-1)

compared to negative control (NALM-6, human B-cell acute lymphoblastic leukemia cell line) and positive control (MCF-7, human breast cancer cell line). **C-D)** CD47 (C) and CD20 (D) surface expression by flow cytometry in MCL cell lines. Tables with *p* values are outlined below corresponding histogram bars (one-way ANOVA with multiple comparisons correction; CD24 *F*(7,16) = 1439, CD47 *F*(8,18) = 1633, CD20 *F*(6,14) = 7526;

experimental triplicate; *****p* < 0.0001).


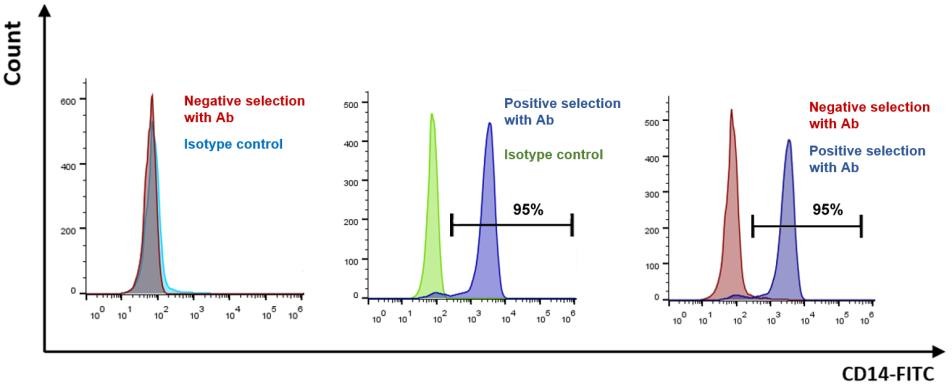
**A**


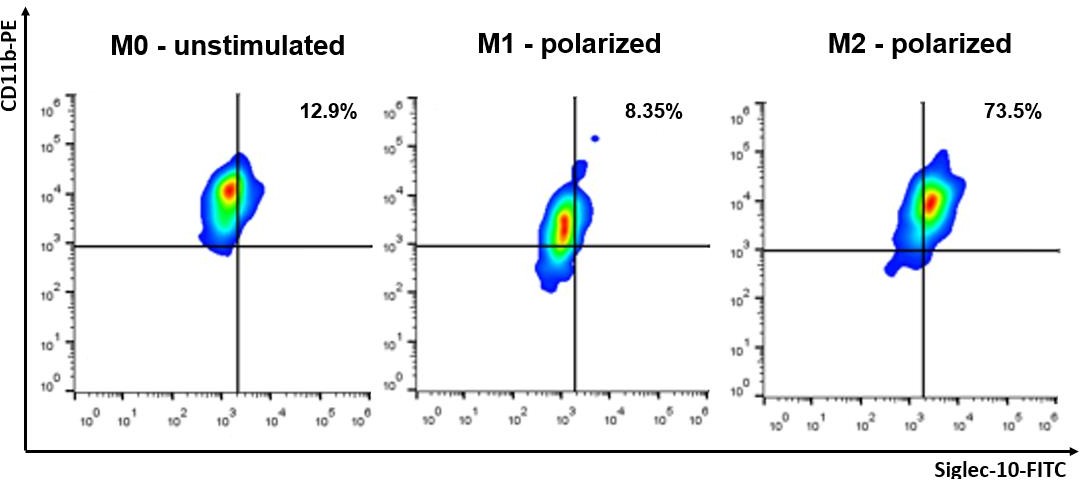
**B**


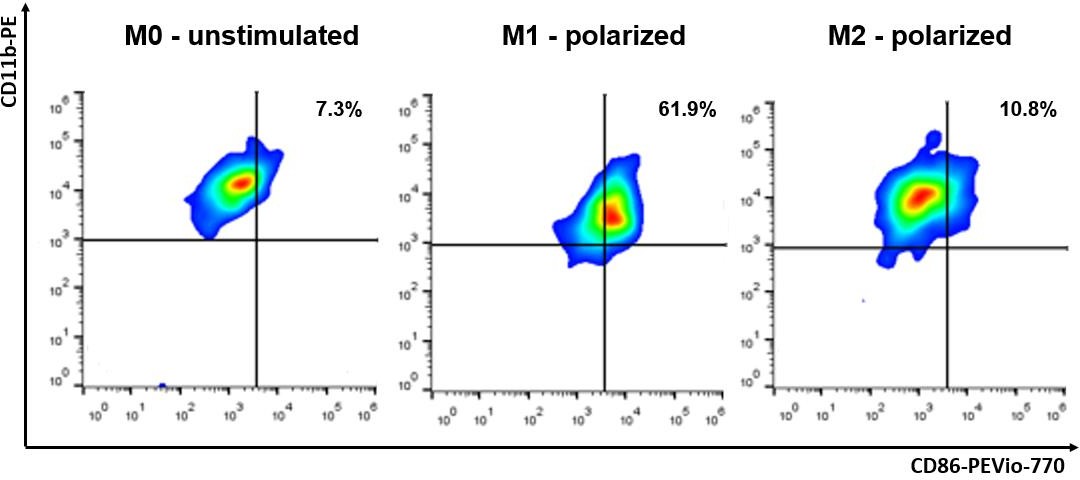

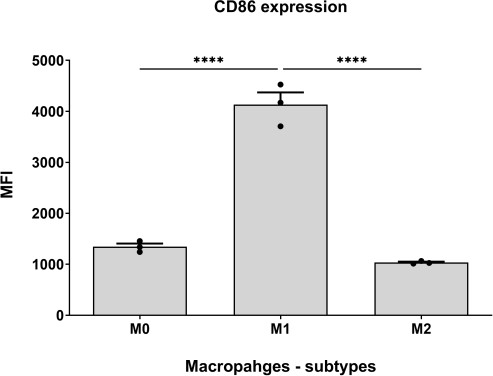
**C
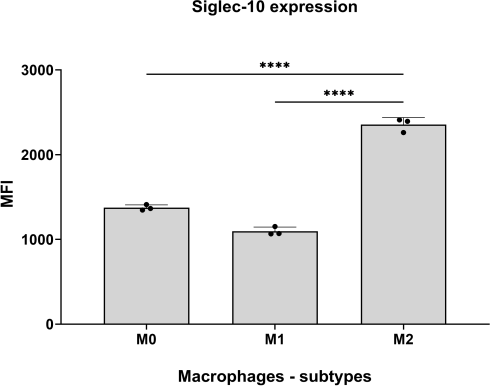
**


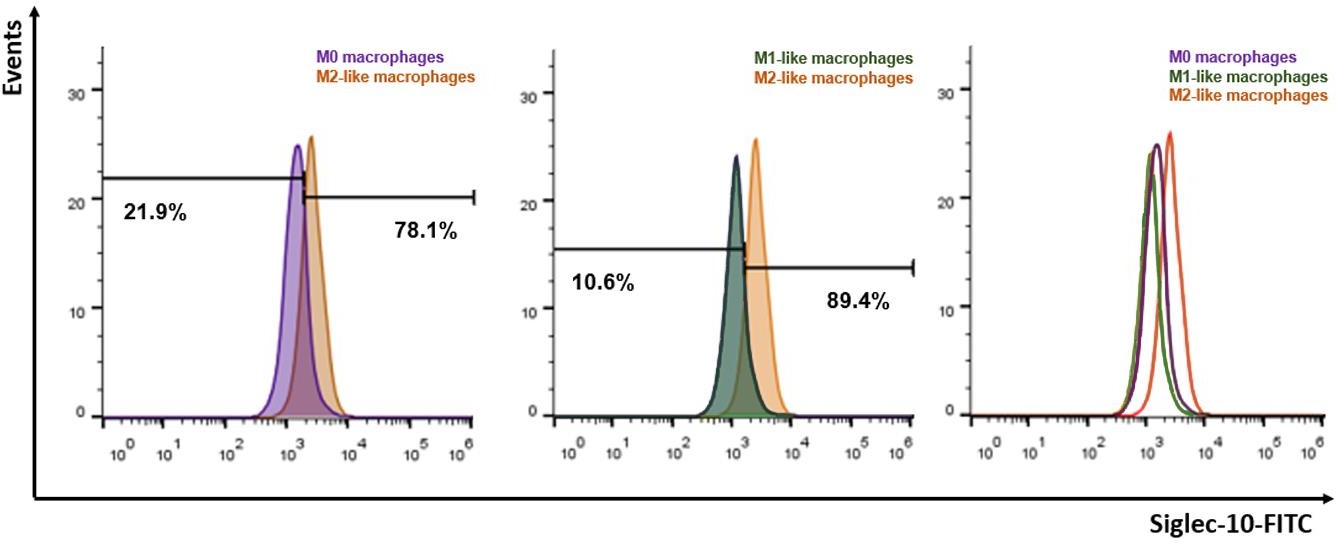
**D**

**
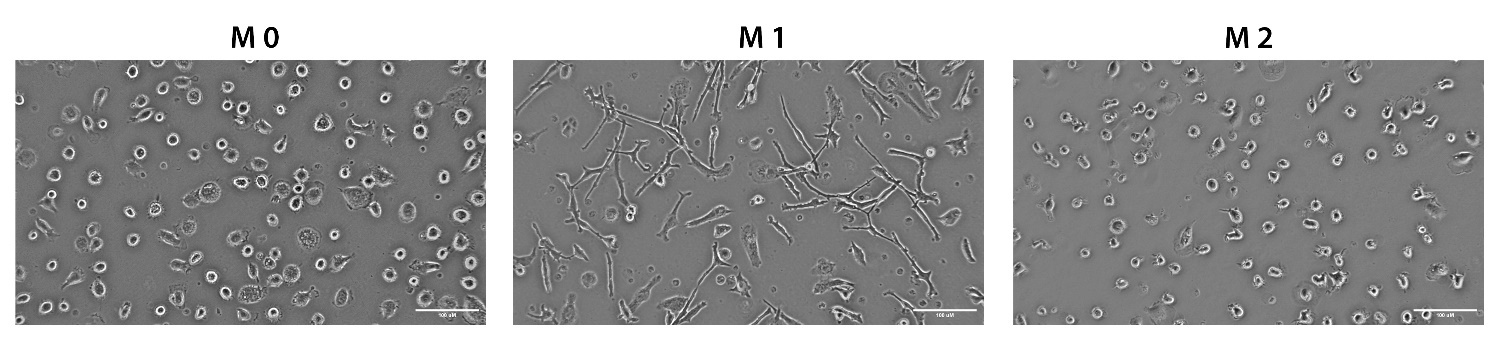
**

**Supplemental Figure 2**

# Supplemental Figure 2

**Monocyte isolation from human PBMC and macrophage differentiation with expression of Siglec-10 as M2-like differentiation marker. A)** Representative histogram after isolation of CD14+ monocytes from PBMC of healthy donors, showing proper separation (> 90%) between negative and positive selection. **B-C)** Macrophage differentiation toward M2- like phenotype, after proper cytokine stimuli (i.e., IL-10, TGF-β_1_), showed higher levels of Siglec-10 if compared to M0 (unstimulated macrophages) and M1-like phenotype; this latter one expressed proper markers of M1 differentiation like CD86 (one-way ANOVA with multiple comparisons correction; Siglec- 10 *F*(2,6) = 381.5, CD86 *F*(2,6) = 144.8; experimental triplicate,

one representative donor; *****p* < 0.0001). **D)** Histogram

overlays for Siglec-10 expression, showing higher levels in M2- like macrophages compared to M0 and M1-like macrophages. Proper cytokines stimuli for each phenotype also conferred modifications in terms of morphology, with star-like shape for M1-like macrophages, in contrast to small round shape for M0 and M2-like macrophages (representative images below histogram overlays).

**A**


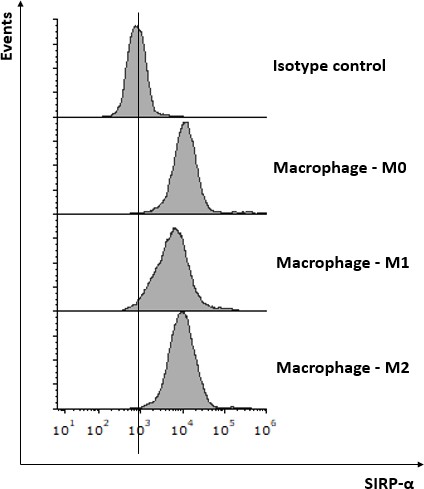

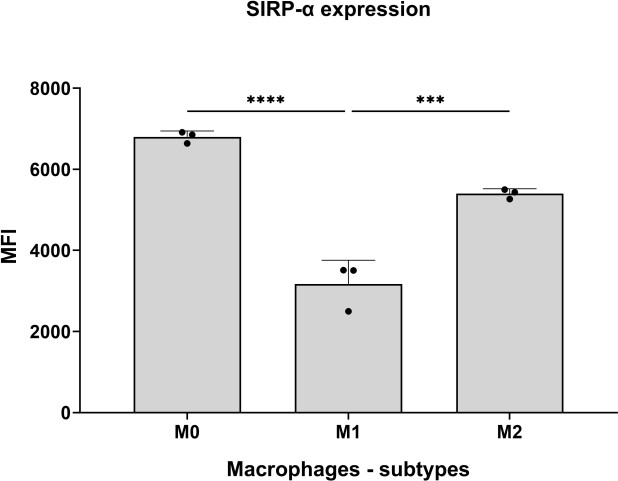


**Supplemental Figure 3**

# Supplemental Figure 3

**SIRP-alpha expression in M2-like macrophages and correlation with other differentiation phenotypes. A)** Representative histogram and histogram bars showing that SIRP-alpha is constitutively expressed in different macrophage phenotypes, with higher expression in M2-like compared to M1-like macrophages (one-way ANOVA with multiple comparisons correction; SIRP-α *F*(2,6) = 80.21; experimental triplicate, one representative donor; ****p* < 0.001, *****p* < 0.0001).

**A**


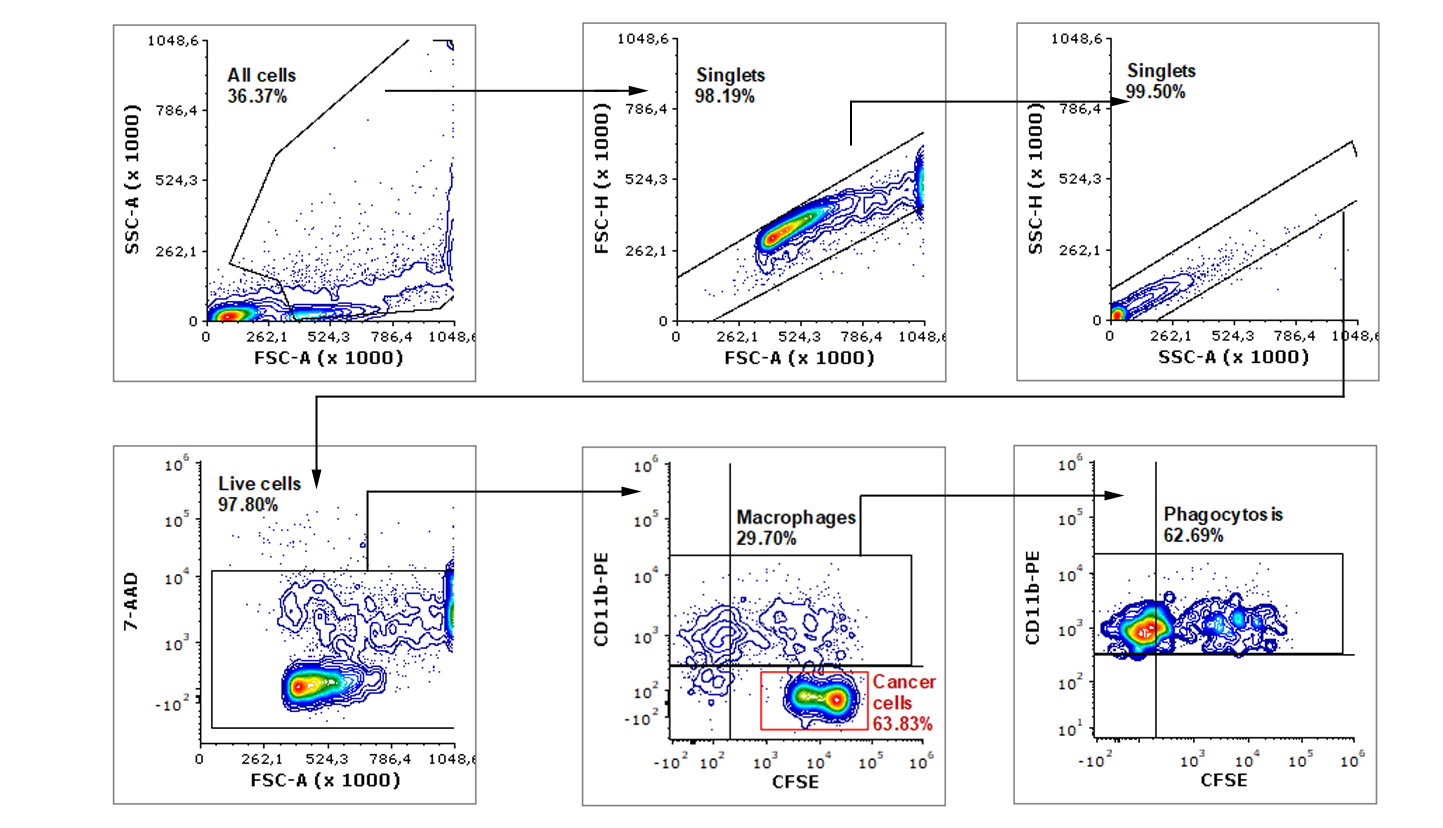


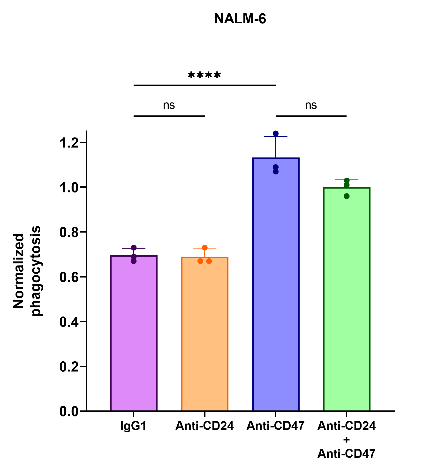
**B**


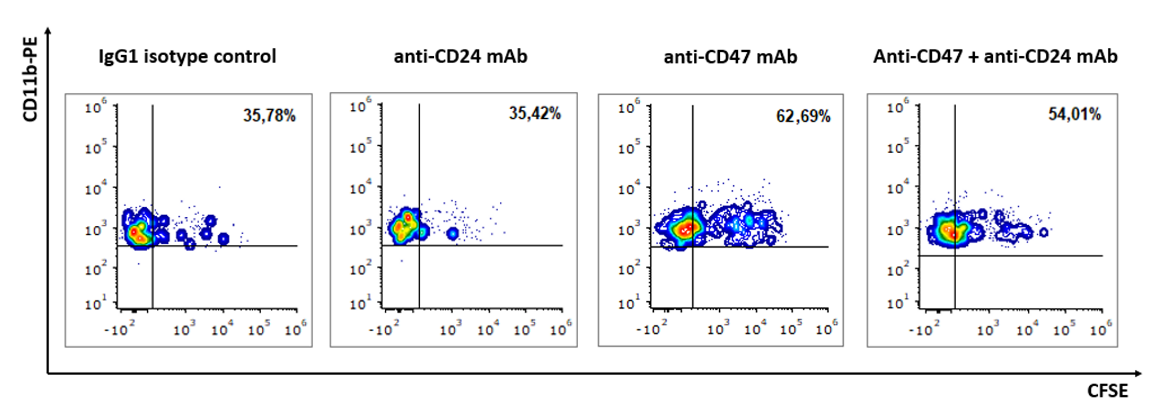


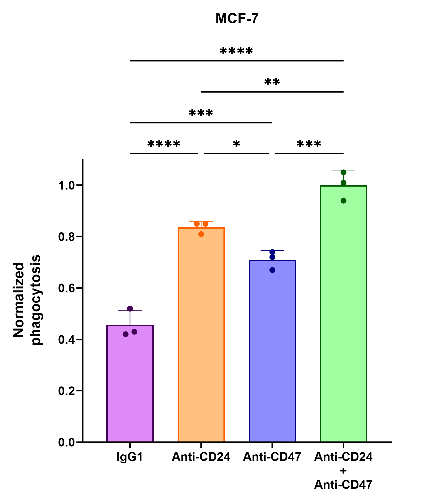


**C**


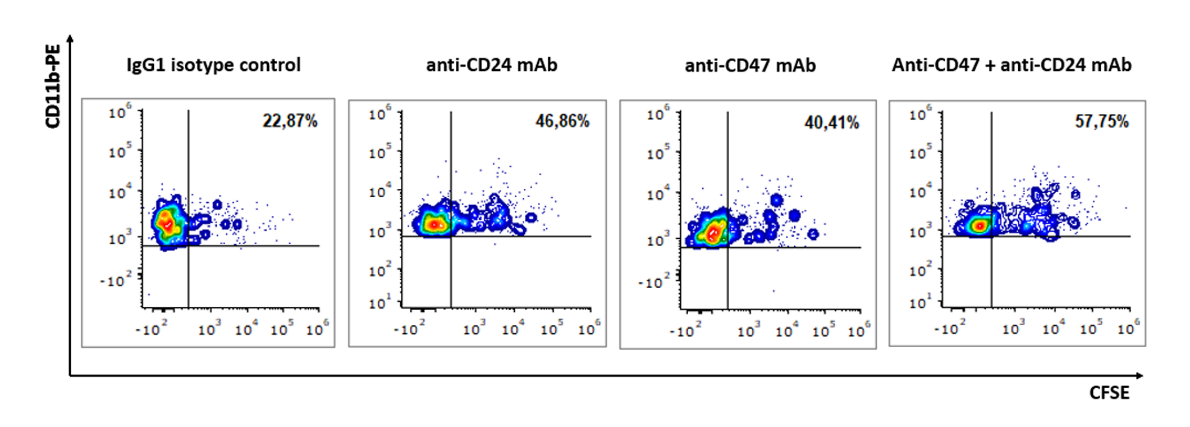


**Supplemental Figure 4**

# Supplemental Figure 4

**Gate strategy for co-culture analysis and testing of phagocytosis in CD24+ and CD24- cancer cell lines after administration of anti-CD24 (clone SN3). A)** To define phagocytosis, gate strategy was performed by ruling out debris and dead cells through morphology and 7-AAD staining, together with doublet removal. Phagocytosis was counted as percentage based on the number of 7-AAD/CD11b+/CFSE+ events out of all 7-AAD-/CD11b+ events (total number of macrophages). Depicted flow cytometry plots were representative of at least 20 experimental replicates and showed co-culture assay with donor-derived M2-like macrophages and NALM-6 cell line (control condition after administration of anti-CD47 mAb). **B-C)** Reproducibility of improvement of phagocytosis of CD24+ cancer cell line (MCF- 7, breast cancer) using anti-CD24 mAb (clone SN3) alone or in combination with anti-CD47 mAb (clone B6H12.2); as expected, anti-CD24 did not improve phagocytosis when M2- like macrophages were co-cultured with CD47+/CD24- cancer cell line (NALM-6), showing increase of phagocytosis only with anti-CD47 administration (one-way ANOVA with multiple comparisons correction; MCF-7 *F*(3,8) = 79.16, NALM-6 *F*(3,8) = 49.16; experimental triplicate, plots from one representative donor; *ns:* not significant, * *p* < 0.05, ** *p* < 0.01, *** *p* < 0.001, **** *p* < 0.0001).


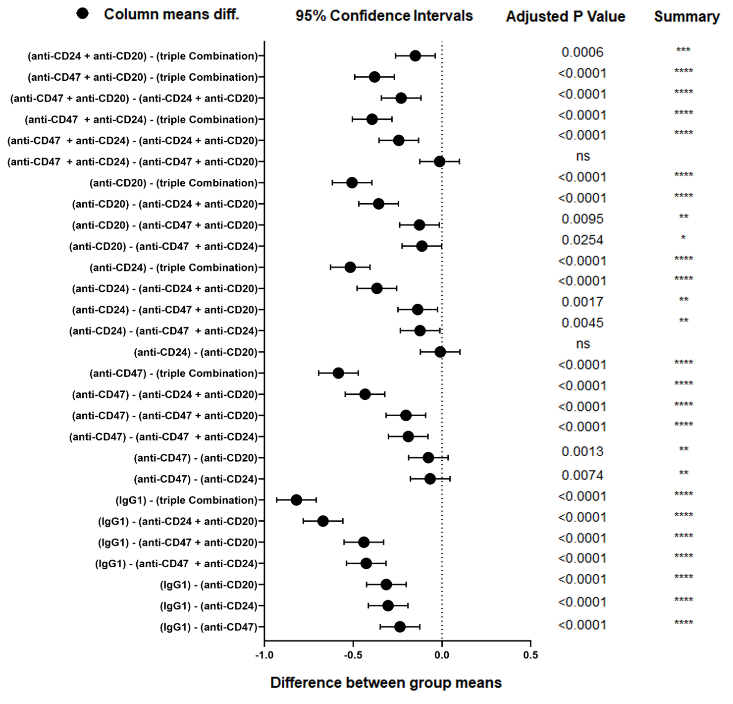

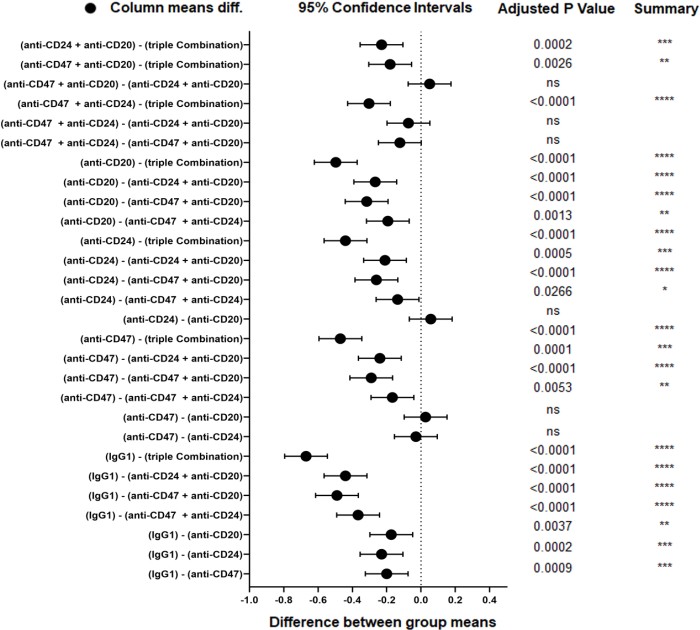


**MINO**


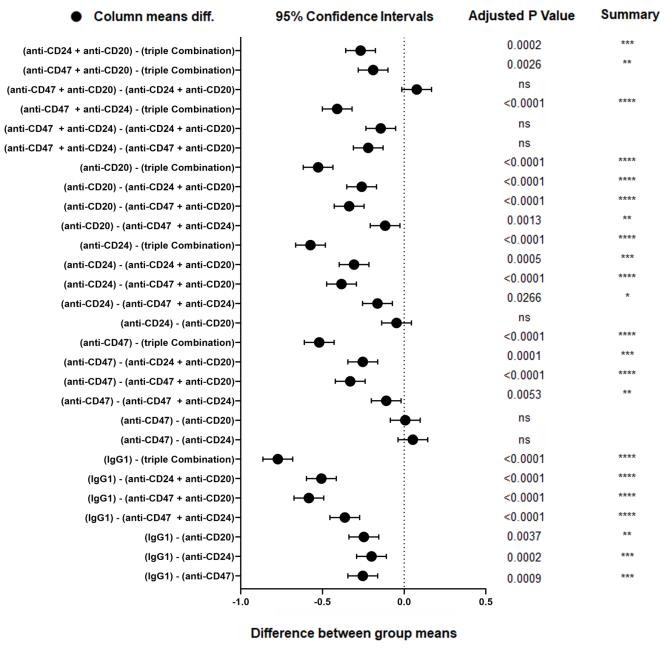


**Granta-519**

**Jeko-1**


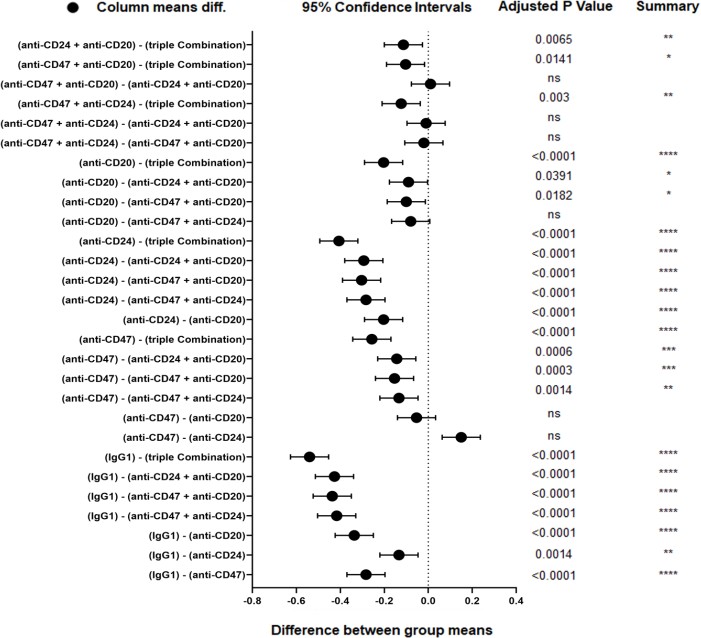


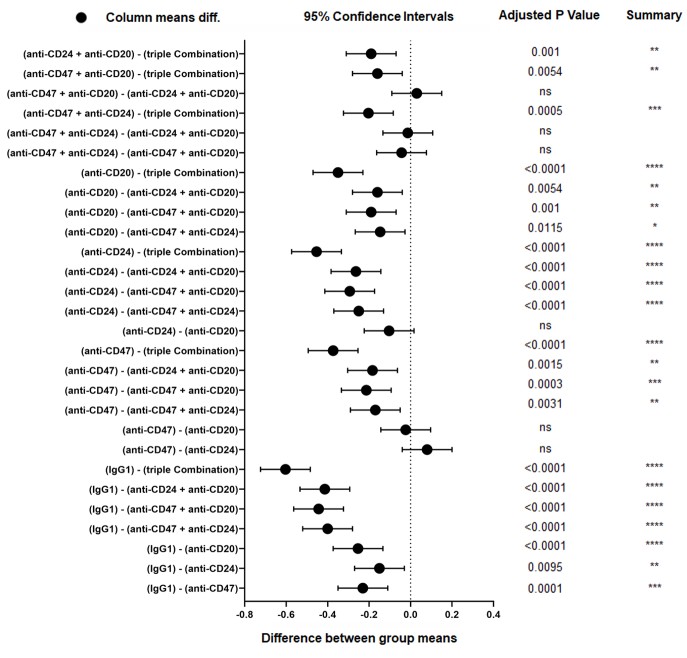
**JVM-2**


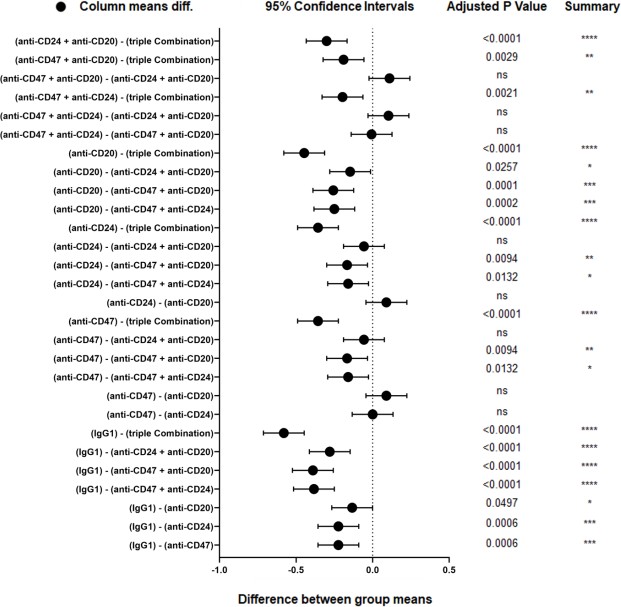


**UPN-1 REC-1**

**Supplemental Figure 5**

# Supplemental Figure 5

**95% Confidence Interval (95% CI) and adjusted *p* value from Tukey’s multiple comparison test for MCL co-culture analysis.**

Recap of multiple comparison tests (Tukey’s method), corresponding 95% CI and adjusted *p* value of the conditions developed after co-culture assays for each MCL cell lines; negative values of differences between group means express statistical significance (one-way ANOVA with multiple comparisons correction; MINO *F*(7,16) = 125.5, Jeko-1 *F*(7,16) = 69.24, Granta-519 *F*(7,16) = 174.1, JVM-2 *F*(7,16) = 100.8, REC-1 *F*(7,16) = 60.31, UPN-1 *F*(7,16) = 42.27; experimental triplicate, *n*= 3 donors; * *p* < 0.05, ** *p* < 0.01, **** *p* < 0.0001).


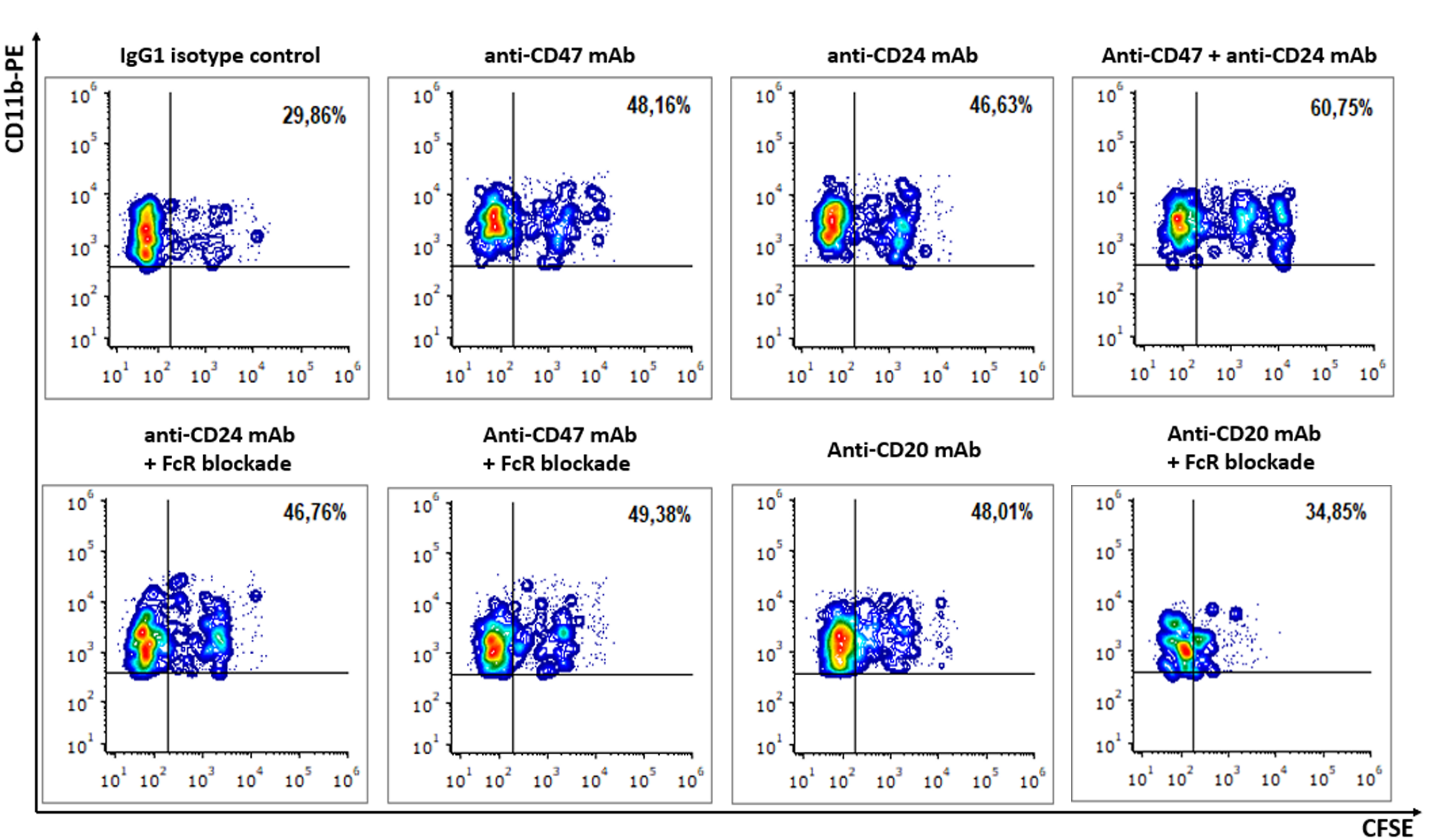
**A**

**B**

**
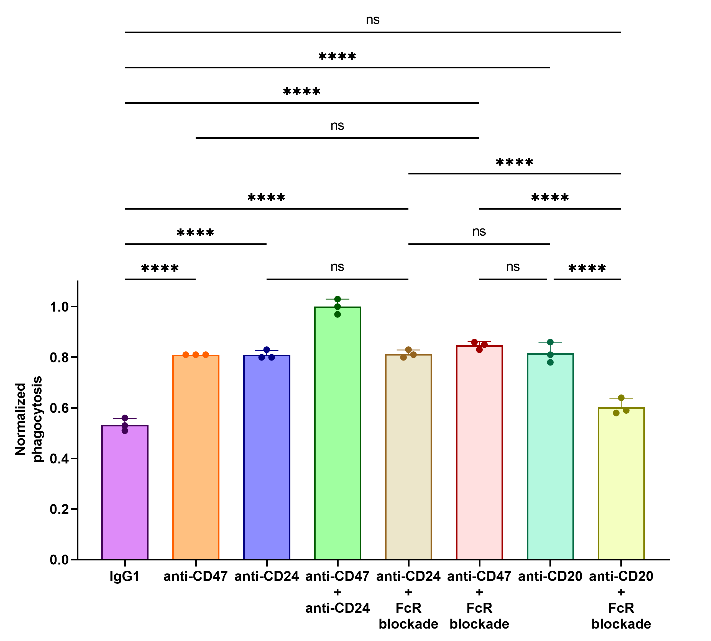

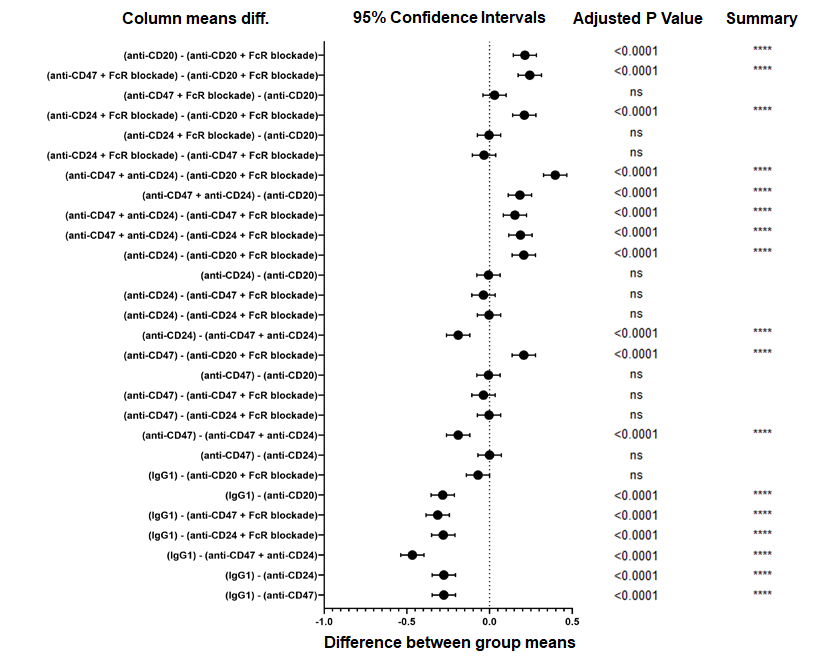
**

**C**


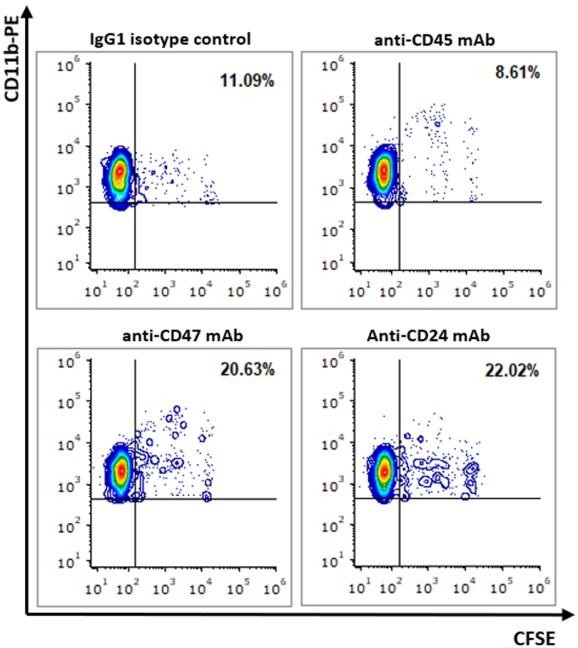


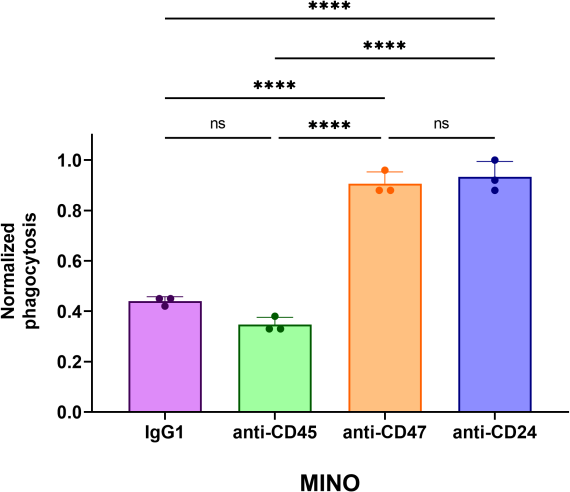
**
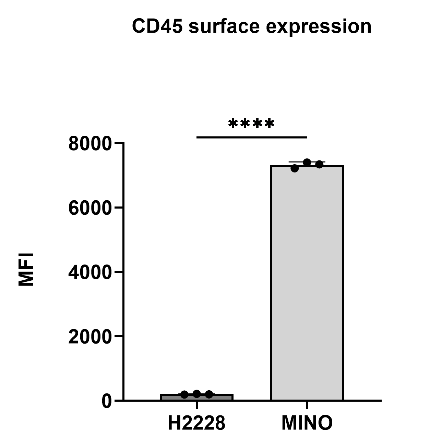
**

**Supplemental Figure 6**

# Supplemental Figure 6

**Improvement of phagocytosis mediated by CD24/Siglec-10 axis blockade rather than Fc-mediated opsonization. A-B)** Representative flow cytometry plot, corresponding histogram bar and 95% CI graph of normalized phagocytosis of MCL cell lines (MINO), showing that untreated M2-like macrophages either with anti-CD24 or anti-CD47 mAbs provided similar phagocytic rate if compared to normalized phagocytosis after use of M2-like macrophages treated with Fc-Receptor (FcR) blocking solution – mixture of anti-CD16, anti-CD32 and anti-CD64 mAbs (anti-CD24 mAb condition with untreated macrophages: blue bar; anti-CD24 mAb condition with macrophages treated with FcR blocking solution: grey-green bar). FcR blocking solution effectively worked since reduction of Rituximab- mediated phagocytosis was documented when treated macrophages are employed (anti-CD20 mAb condition with untreated macrophages: light blue bar; anti-CD20 mAb condition with macrophages treated with FcR blocking solution: yellow bar. One-way ANOVA with multiple comparisons

correction; MINO *F*(7,16) = 103; *n* = 2 donors; representative technical triplicate of one experimental cohort; *ns*: not significant; **** *p* < 0.0001). **C)** CD45 MFI showed higher expression in MINO if compared to H2228 (human lung cancer cell line), since CD45 is a surface antigen that is pan-expressed by hematopoietic-derived cells. Histogram bars and representative flow cytometry plot of co-culture assay with M2-like macrophages and MCL cell line (MINO) showed that anti-CD45 mAb provided minor phagocytosis if compared to anti-CD24 and anti-CD47 mAbs, still indicating that phagocytosis after administration of anti-CD24 mAb is secondary to loss of CD24 signalling rather than to Fc-mediated opsonization (CD45 MFI: unpaired, two-tailed Student’s *t*-test, **** *p* < 0.0001; co-culture assay: one-way ANOVA with multiple comparisons correction; MINO *F*(3,8) = 161.2; experimental triplicate, plots from one representative donor; *ns:* not significant, **** *p* < 0.0001).


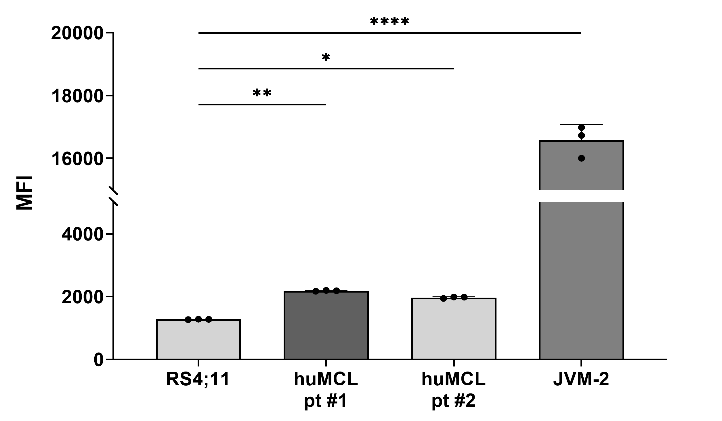


**CD47 surface expression**

**CD24 surface expression**


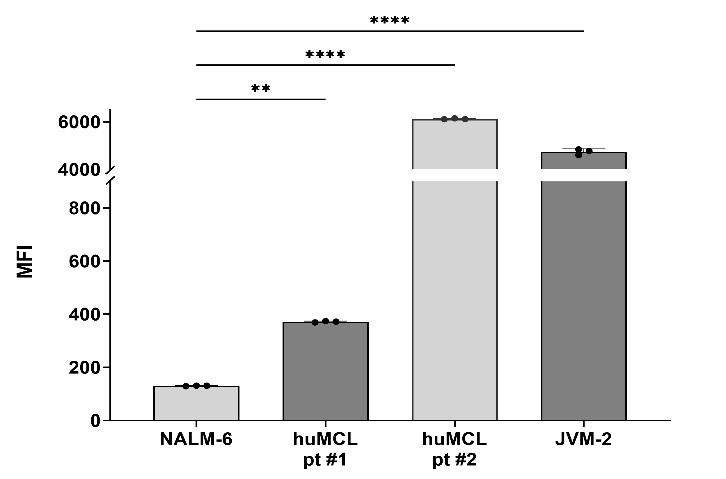


**A**

**B**


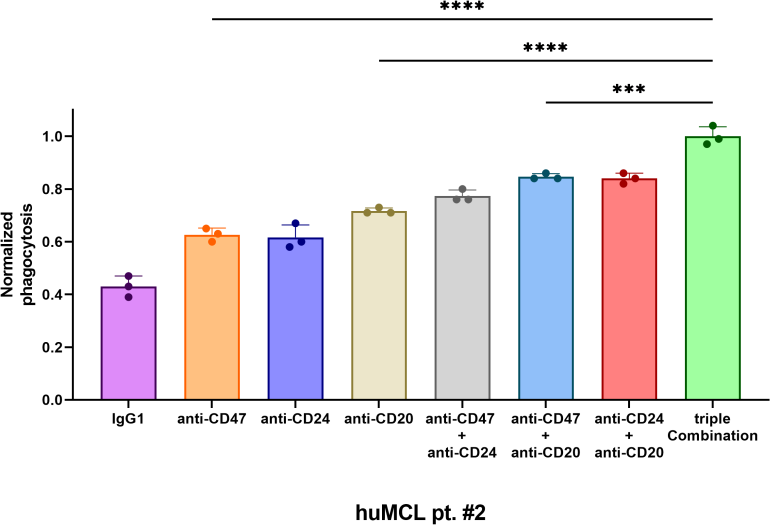

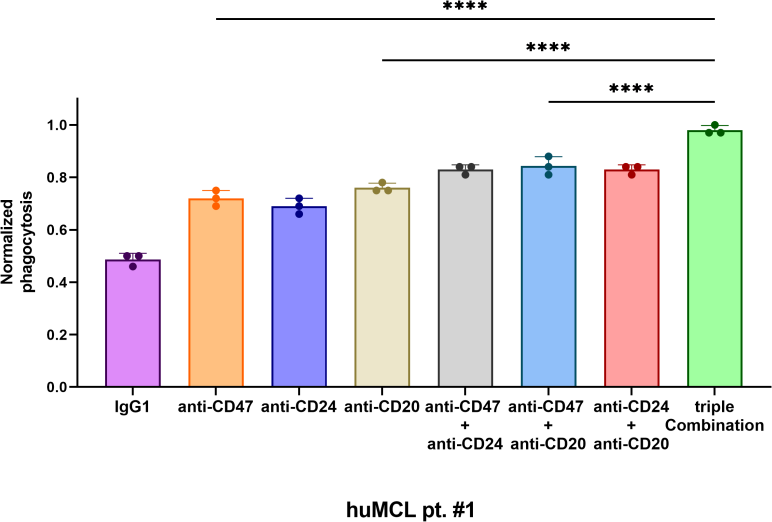


**Supplemental Figure 7**

**D**


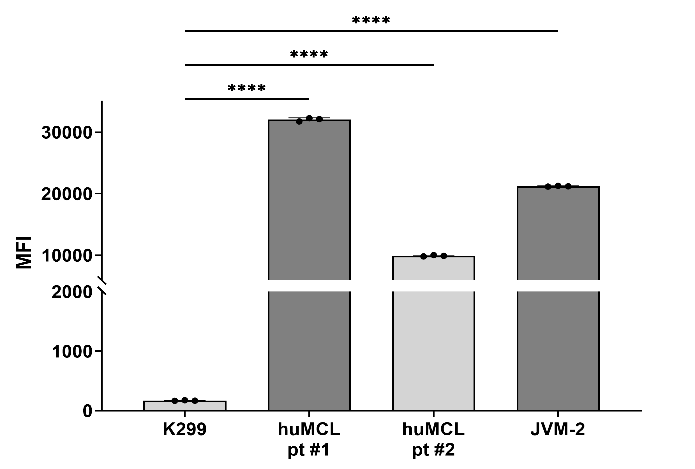


**CD20 surface expression**

**E**

**C**

# Supplemental Figure 7

**Reproducibility of increased phagocytosis after co- culturing MCL patient-derived samples presenting CD24low- density and CD24high-density expression together with M2-like macrophages. A-C)** Histogram bars of MCL patient-derived sample with CD24low-density and CD24high-density expression (A, huMCL pt. #1 with higher expression than NALM-6 but lower than JVM-2; huMCL pt. #2 with higher expression than JVM-2), and corresponding CD47 and CD20 surface expression (B-C, one-way ANOVA with multiple comparisons correction; CD24 *F*(2,6) = 4128, CD47 *F*(3,8) = 2527, CD20 *F*(3,8) = 25982; experimental triplicate; * *p* < 0.05, ** *p* < 0.01, **** *p*< 0.0001). **D-E)** Normalized phagocytosis of huMCL confirmed increase of phagocytosis when DEMs blockade deployed in co-culture experiments, with highest phagocytosis rate in case of triple Combination (anti-CD47 + anti-CD20 + anti-CD24 mAbs. One-way ANOVA with multiple comparisons correction; huMCL pt. #1 *F*(7,16) = 104.9, huMCL pt. #2 *F*(7,16) = 105.4; technical triplicate, one representative donor; *** *p* < 0.001, **** *p*< 0.0001).


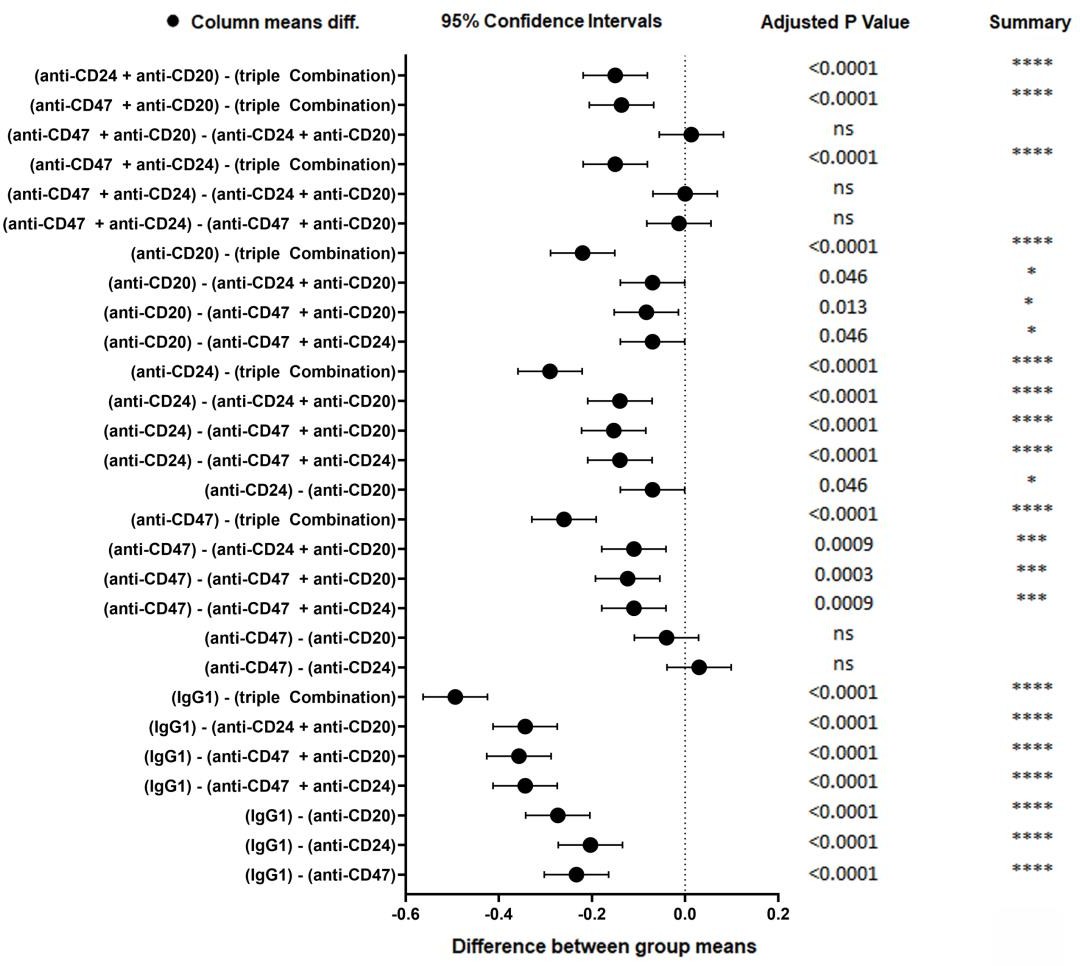
**A**


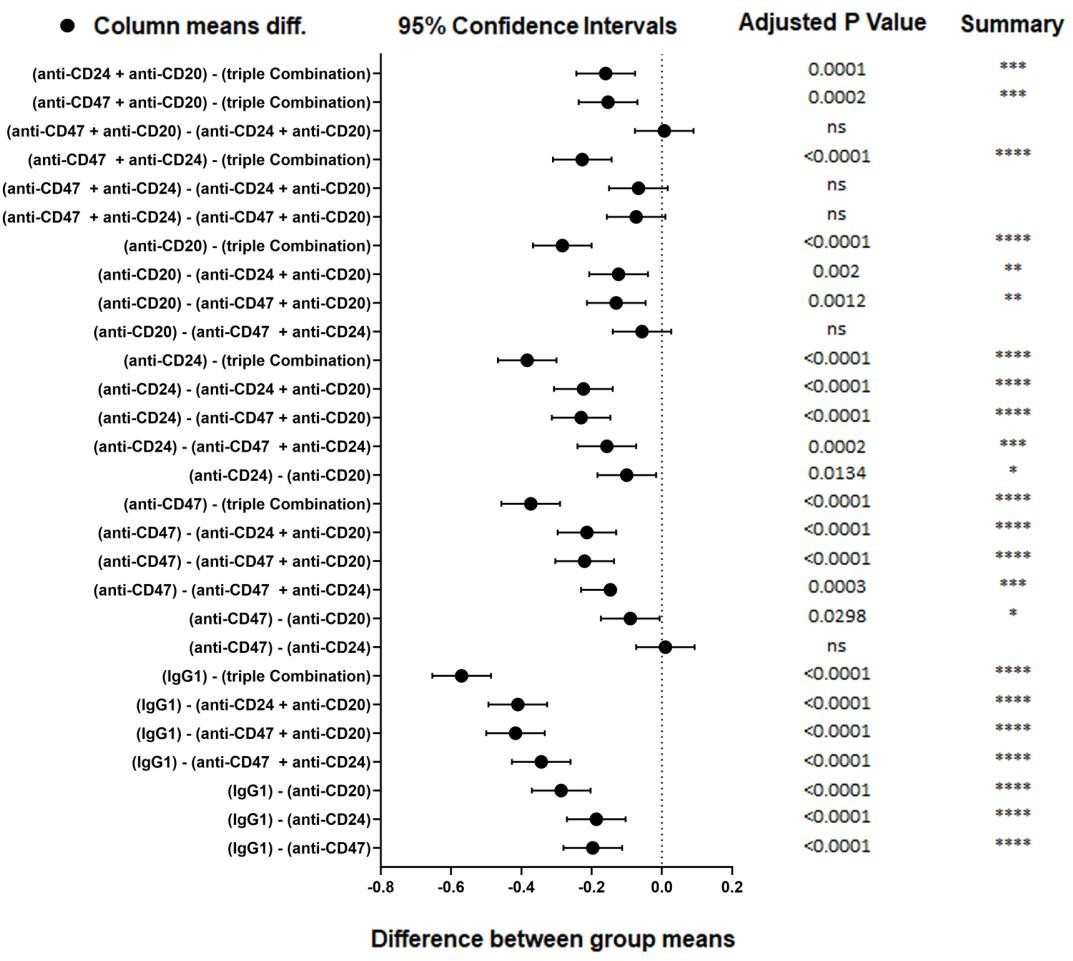
**huMCL pt. #1**

**B**

**huMCL pt. #2**

**Supplemental Figure 8**

# Supplemental Figure 8

**95% Confidence Interval (95% CI) and adjusted *p* value from Tukey’s multiple comparison test for huMCL co- culture analysis. A-B)** Recap of multiple comparison tests (Tukey’s method), corresponding 95% CI and adjusted *p* value of the conditions developed after co-culture assays for huMCL patient-derived samples; negative values of differences between group means express statistical significance (one-way ANOVA with multiple comparisons correction; huMCL pt. #1 *F*(7,16) = 104.9, huMCL pt. #2 *F*(7,16) = 105.4; technical triplicate, one representative donor; *ns*: not significant; * *p* < 0.05, ** *p* < 0.01, *** *p* < 0.001, **** *p* < 0.0001).


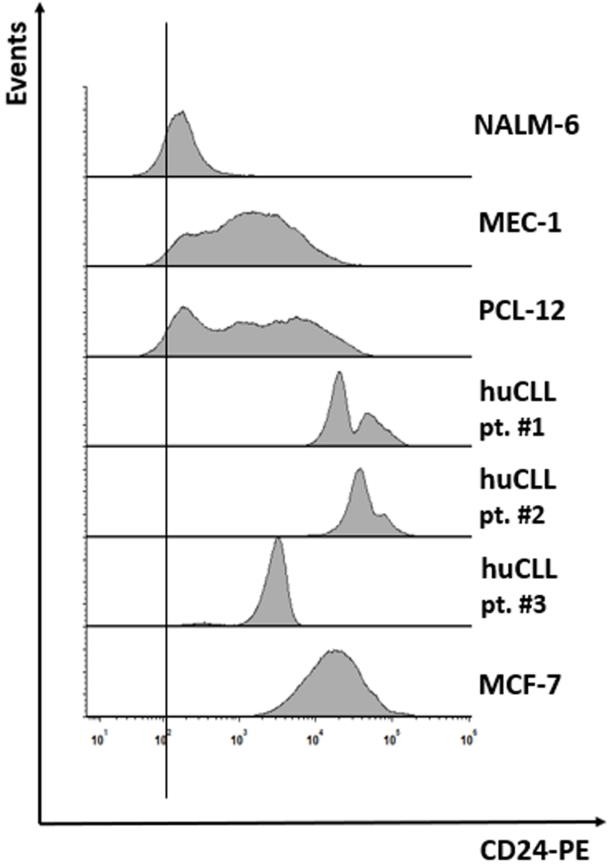
**A**


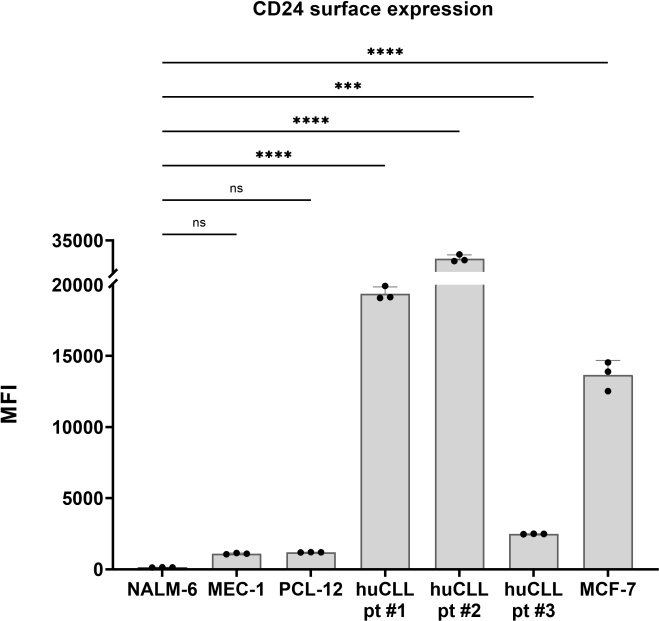


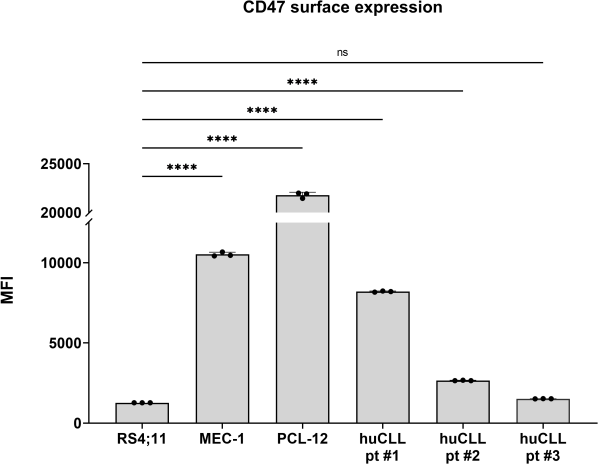

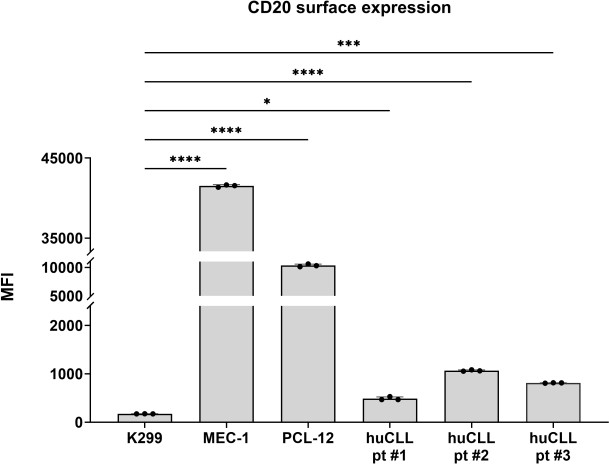
**B**


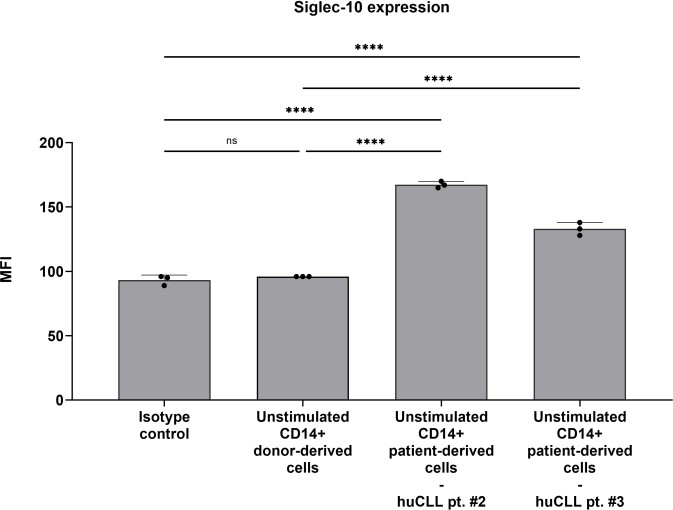
**C**

**Supplemental Figure 9**

# Supplemental Figure 9

**CD24, CD47 and CD20 upregulation in CLL cell lines and patients-derived samples, as well as Siglec-10 expression in patient-derived unstimulated CD14+ cells (monocyte- macrophage system). A-B)** Representative flow cytometry histogram showing that CD24 upregulation was found in patients-derived samples (huCLL pt. #1, huCLL pt. #2, huCLL pt. #3), whereas CLL cell lines provided lower and heterogeneous expression (histogram bar, right panel). CD47 and CD20 markers were lower – but trendily significant – expressed in patients-derived samples (one-way ANOVA with multiple comparisons correction; experimental triplicate, *ns*: not significant, * *p* < 0.05, *** *p* < 0.0001, **** *p* < 0.0001). **C)** Siglec-10 expression was tested and turned out to be higher in CD14+ patient-derived cells from huCLL pt. #2 and huCLL pt. #3 (circulating monocyte-macrophagic cells) with respect to donor-derived counterpart and isotype control (one-way ANOVA with multiple comparisons correction; CD24 *F*(6,14) = 1972, CD47 *F*(5,12) = 10985, CD20 *F*(5,12) = 57592, Siglec-10 *F*(3,8) = 321.7; technical triplicate, one representative donor; *ns*: not significant, **** *p* < 0.0001)**.**

**A**


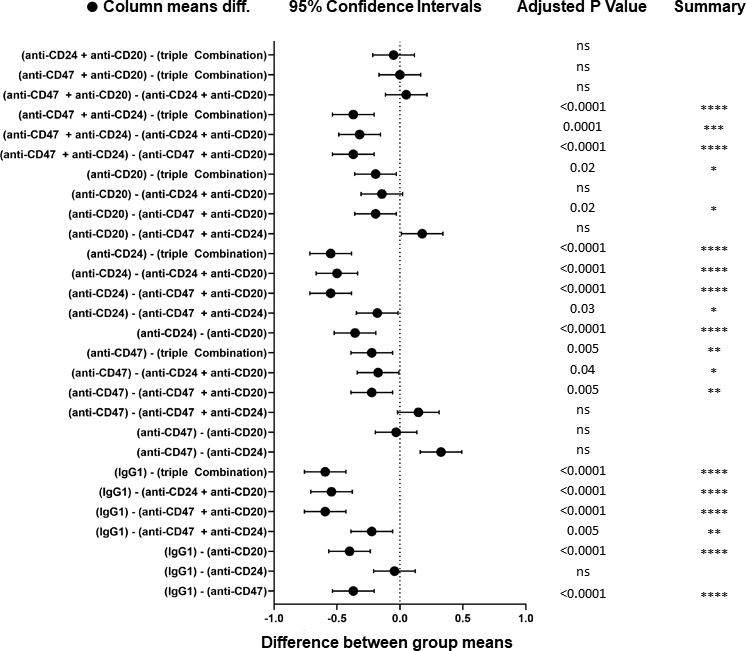

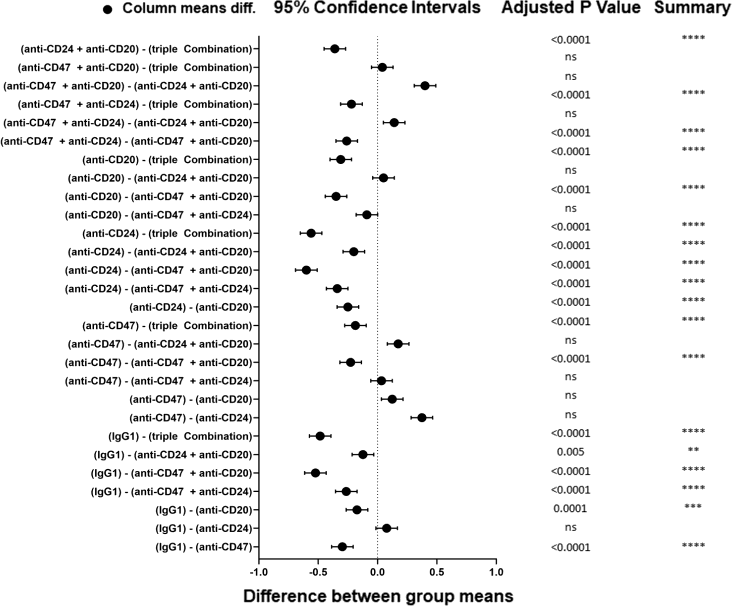


**MEC-1 PCL-12**

**B**


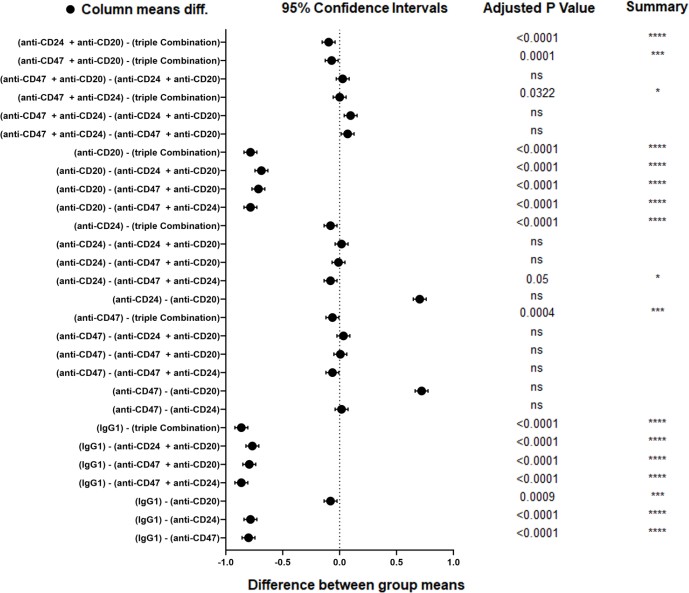

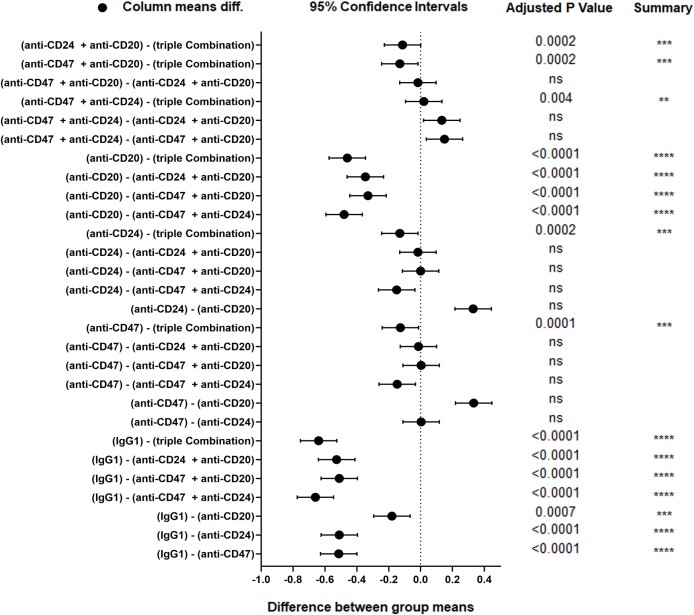


**huCLL pt. #1 – donor Macrophages huCLL pt. #1 – autologous CD14+ cells**

**Supplemental Figure 10**


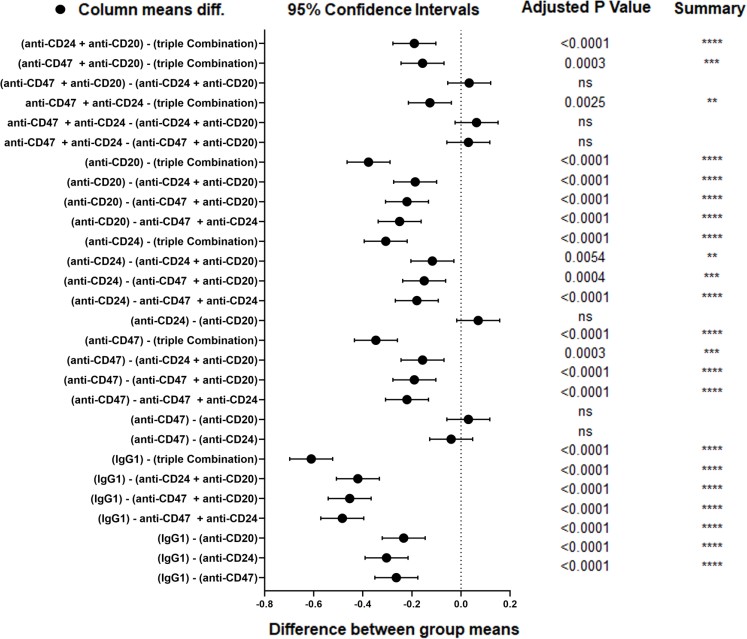
**C**


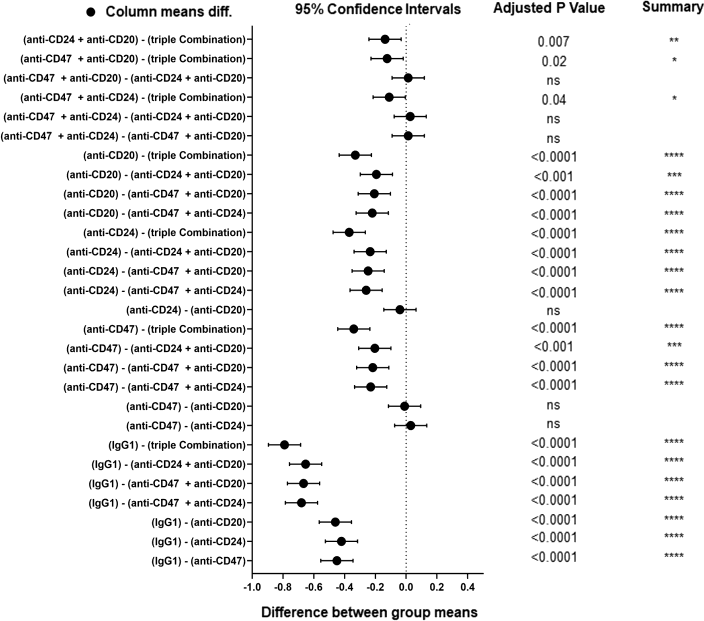


**huCLL pt. #2 – donor Macrophages huCLL pt. #2 – autologous CD14+ cells**


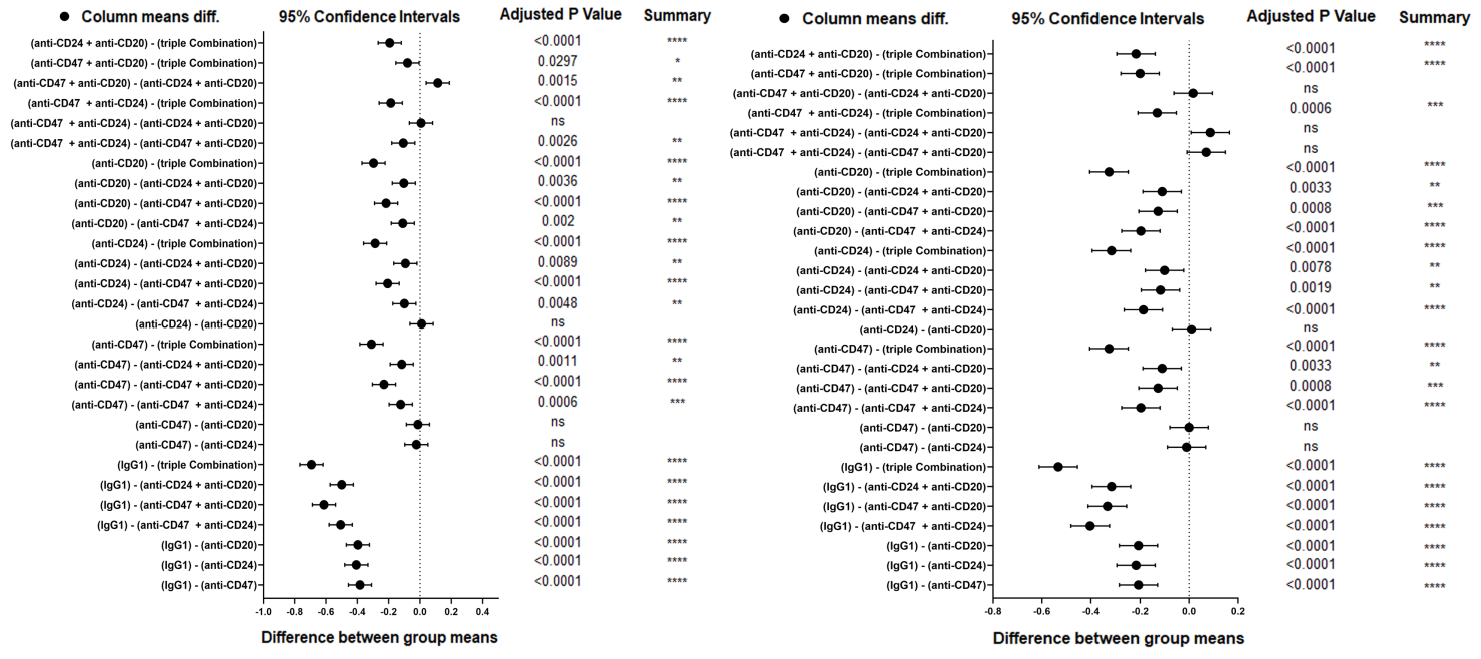
**D**

**huCLL pt. #3 – donor Macrophages huCLL pt. #3 – autologous CD14+ cells**

**Supplemental Figure 10**

# Supplemental Figure 10

**95% Confidence Interval (95% CI) and adjusted *p* value from Tukey’s multiple comparison test for CLL cell lines and patients-derived samples. A-D)** Recap of multiple comparison tests (Tukey’s method), corresponding 95% CI and adjusted *p* value of the conditions developed after co-culture assays for CLL cell lines and patients-derived CLL samples; negative values of differences between group means express statistical significance (one-way ANOVA with multiple comparisons correction; MEC-1 *F*(7,16) = 49.12, PCL-12 *F*(7,16) = 131.5, huCLL pt. #1 w/ donor-MØ *F*(7,16) = 1164, huCLL pt. #1 w/auto-MØ *F*(7,16) = 110.6, huCLL pt. #2 w/ donor-MØ *F*(7,16) = 133.3, huCLL pt. #2 w/ auto-MØ *F*(7,16) = 110.3, huCLL pt. #3 w/ donor-MØ *F*(7,16) = 188.3, huCLL pt. #3 w/ auto-MØ *F*(7,16) = 98.76; technical triplicate, one representative donor; *ns*: not significant, * *p* < 0.05, *** *p* < 0.001, **** *p* < 0.0001).


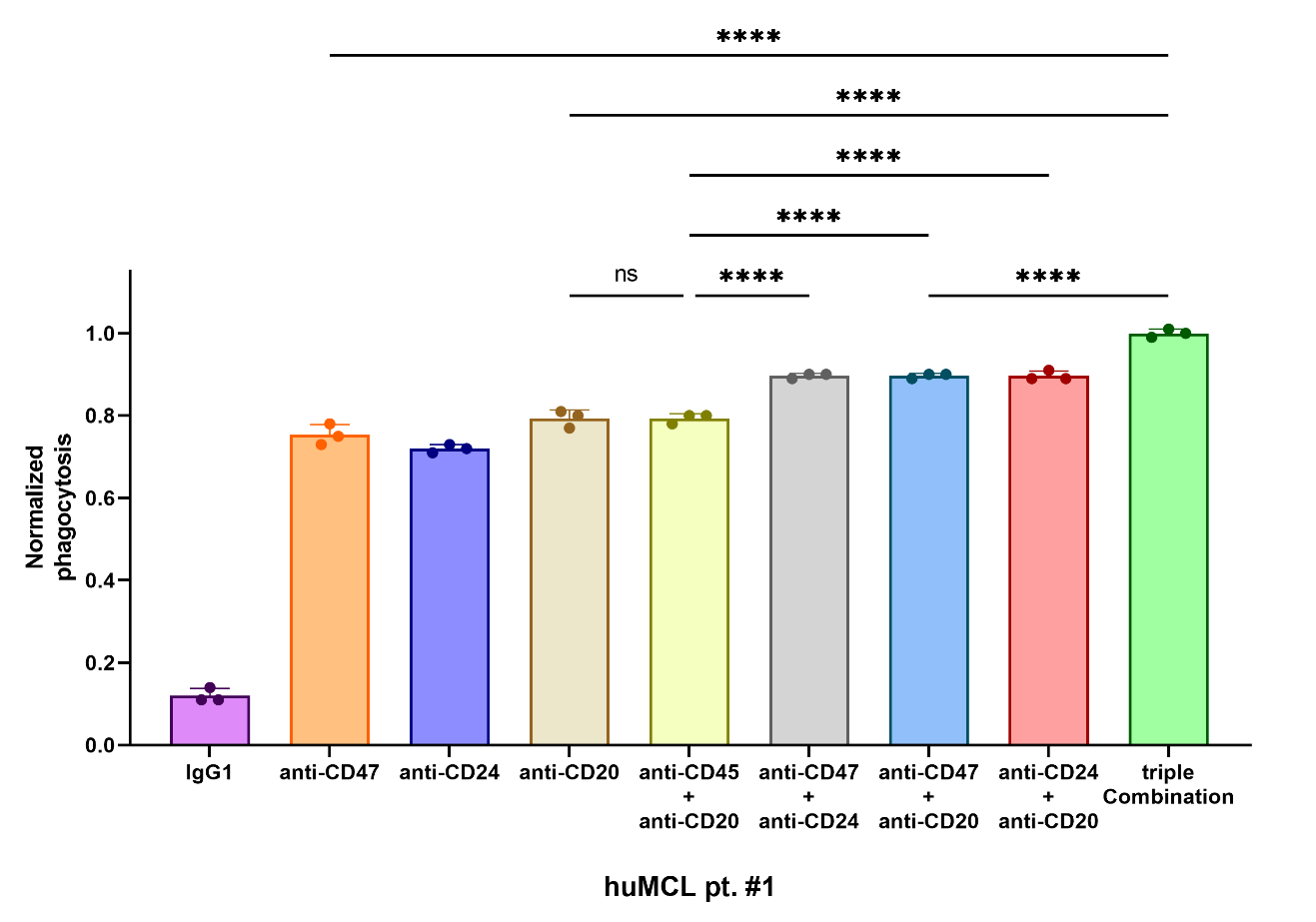

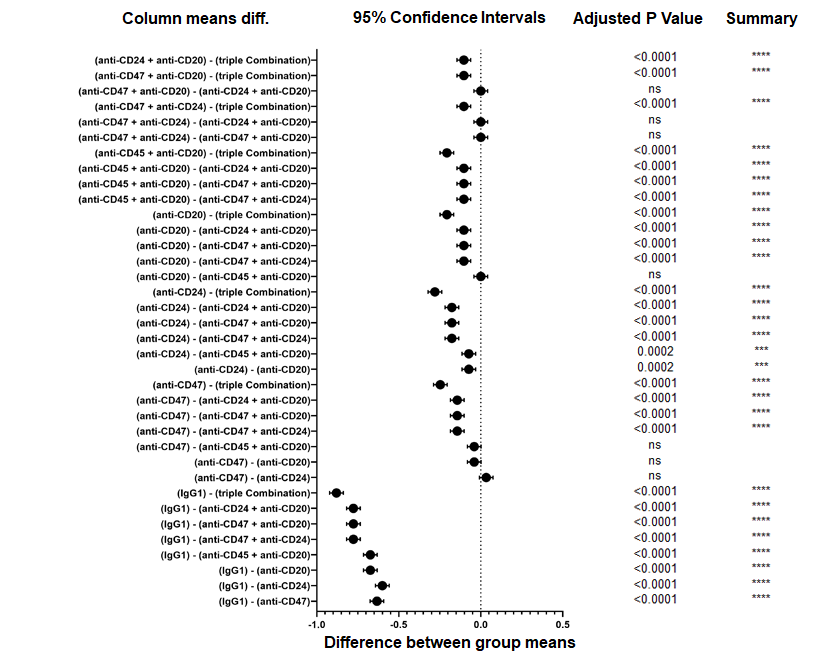


**Supplemental Figure 11**

**A**


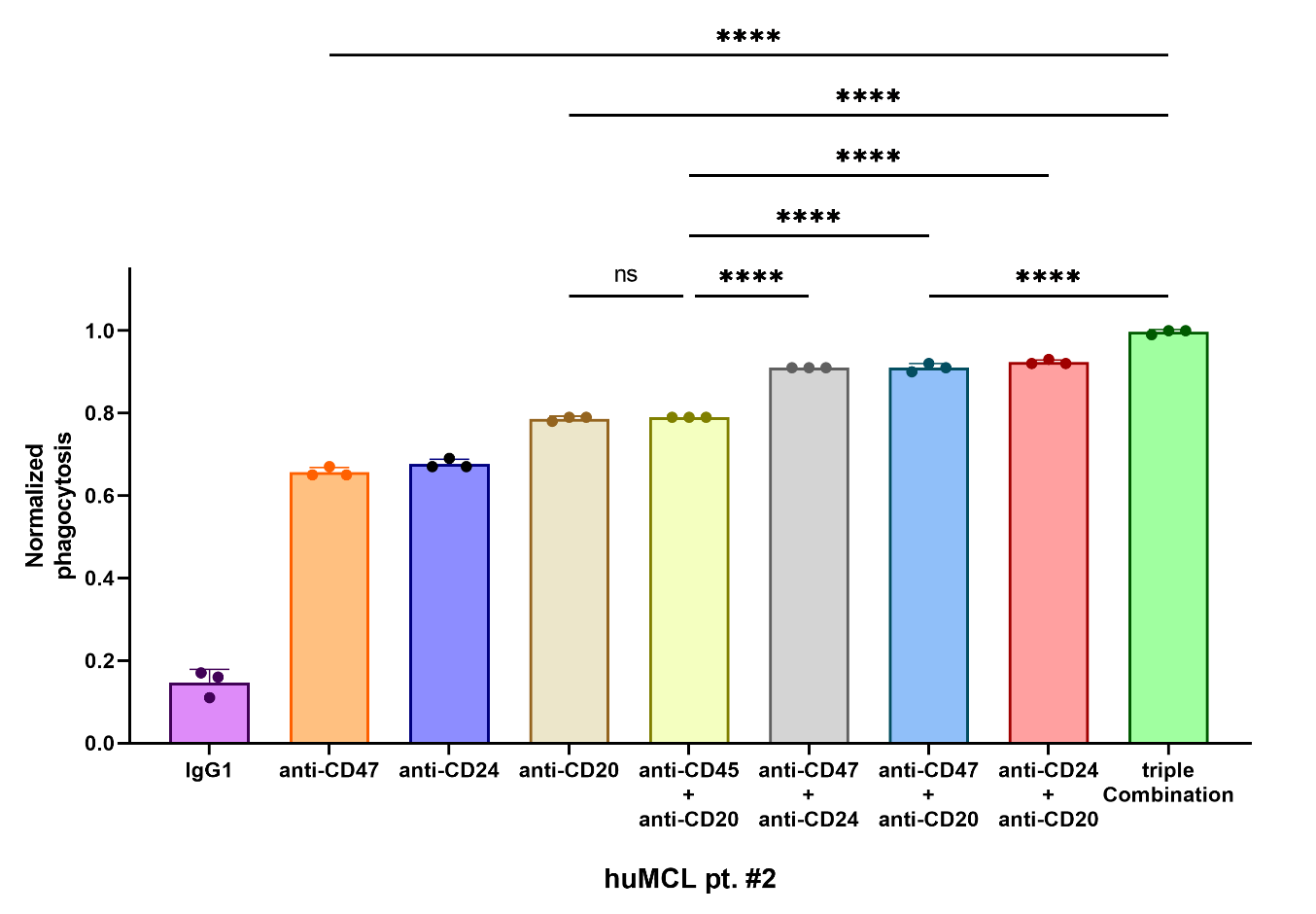


**B**


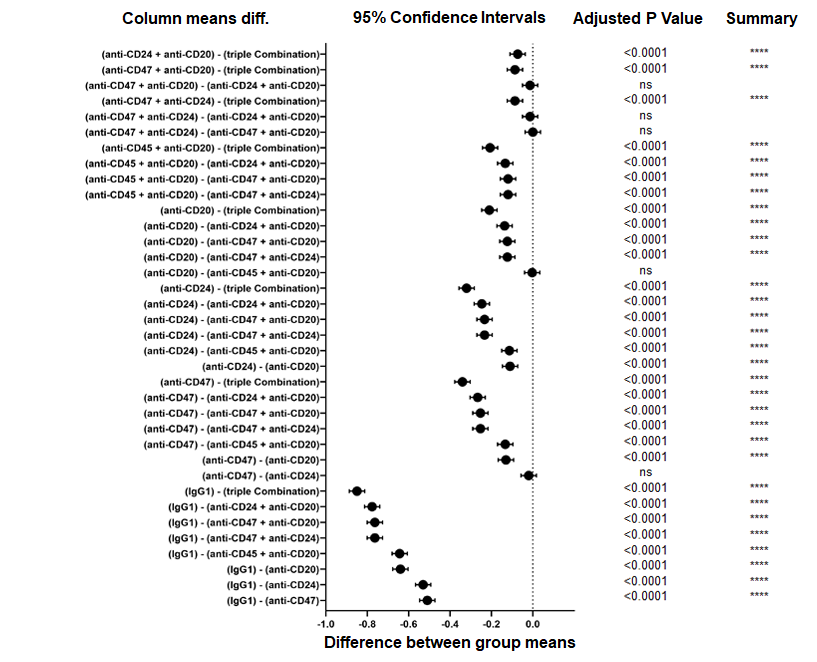


**Supplemental Figure 11**


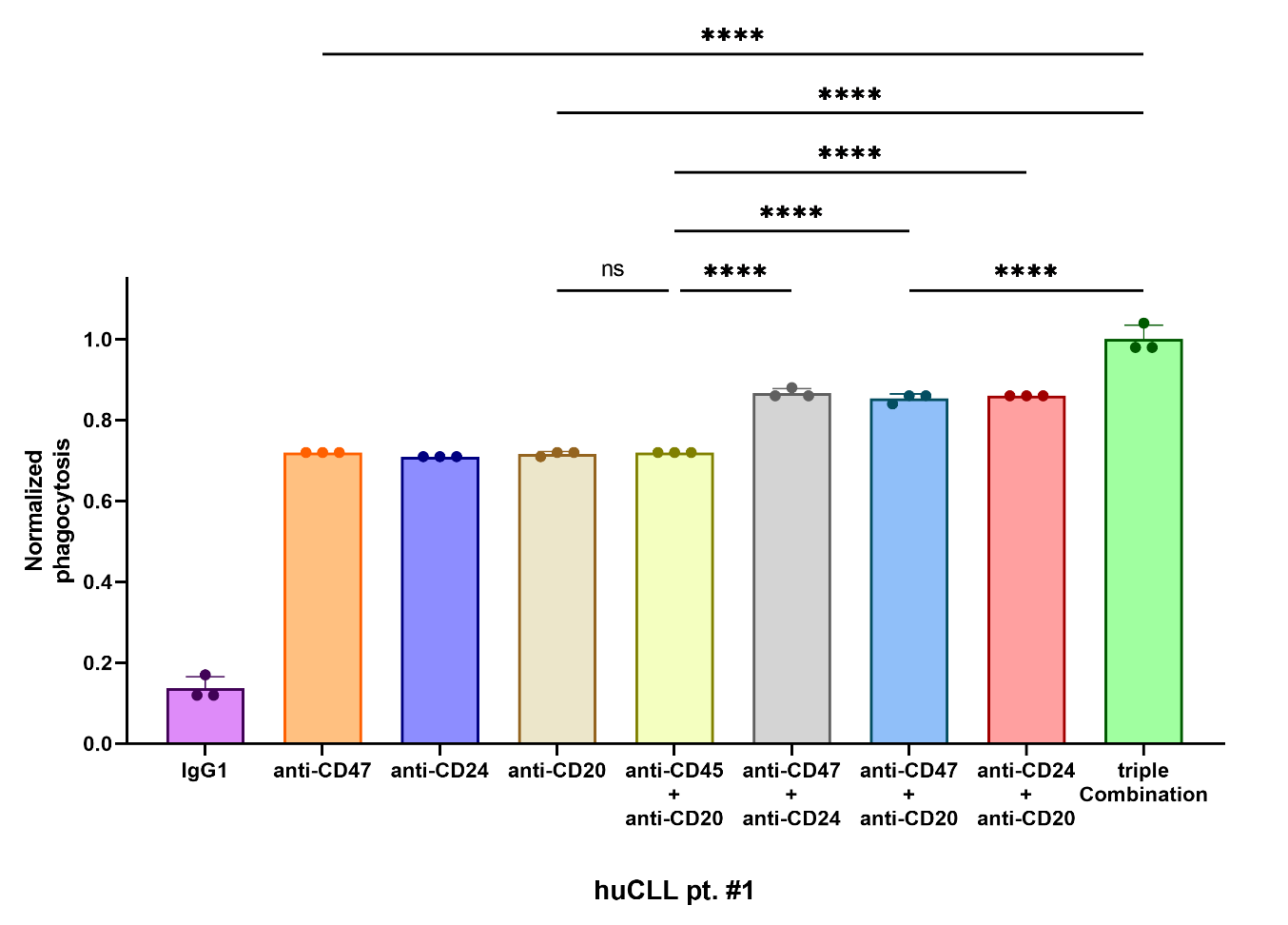


**C**


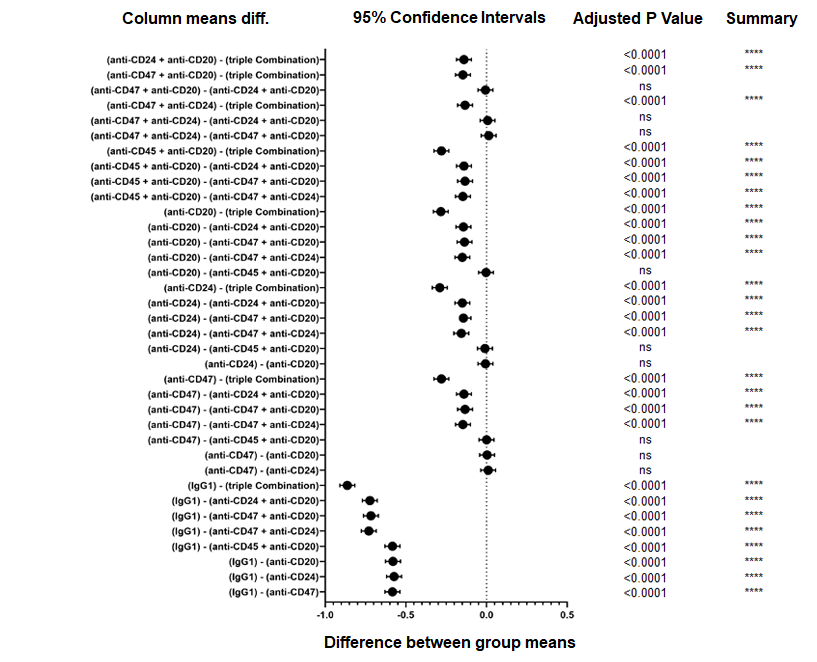


**Supplemental Figure 11**


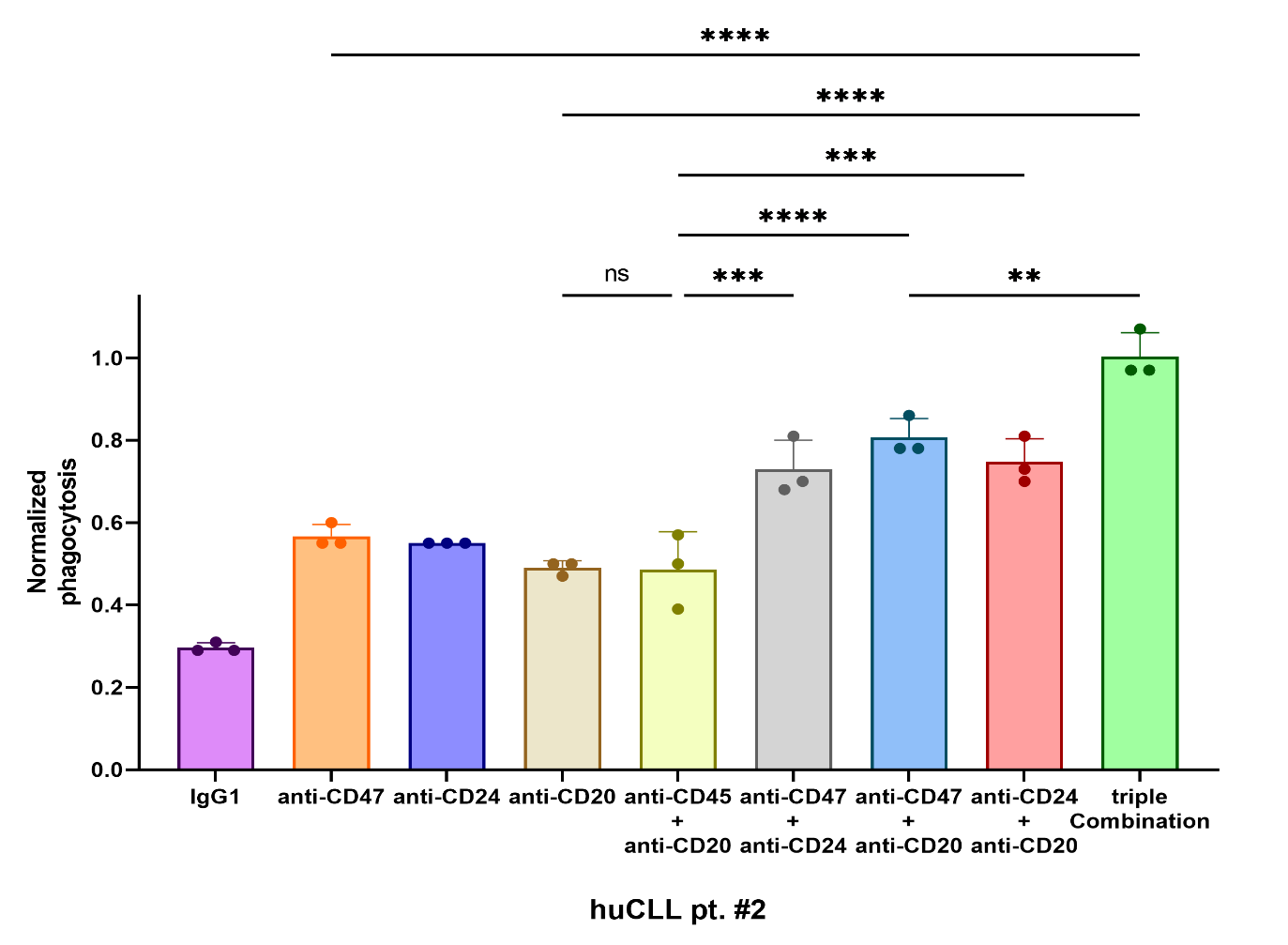


**D**


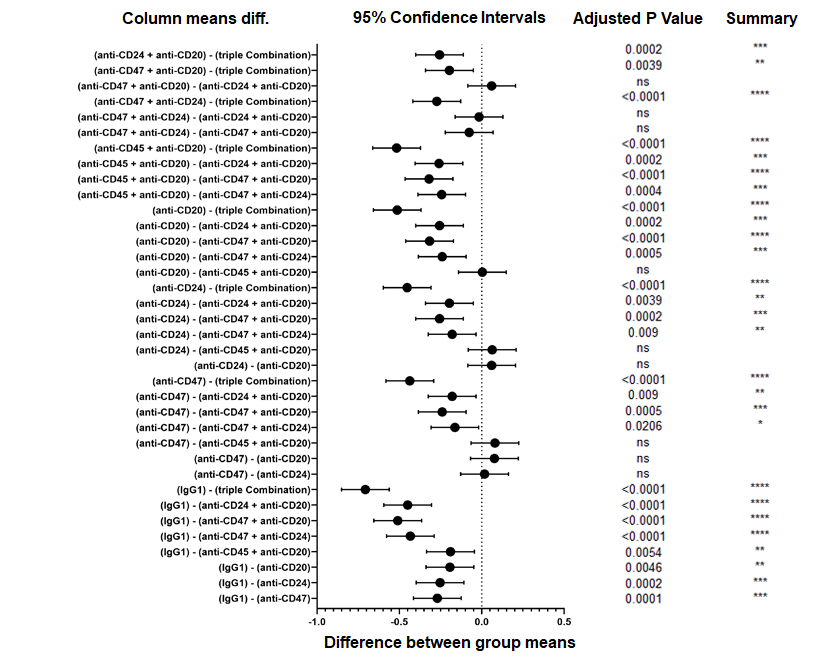


**Supplemental Figure 11**


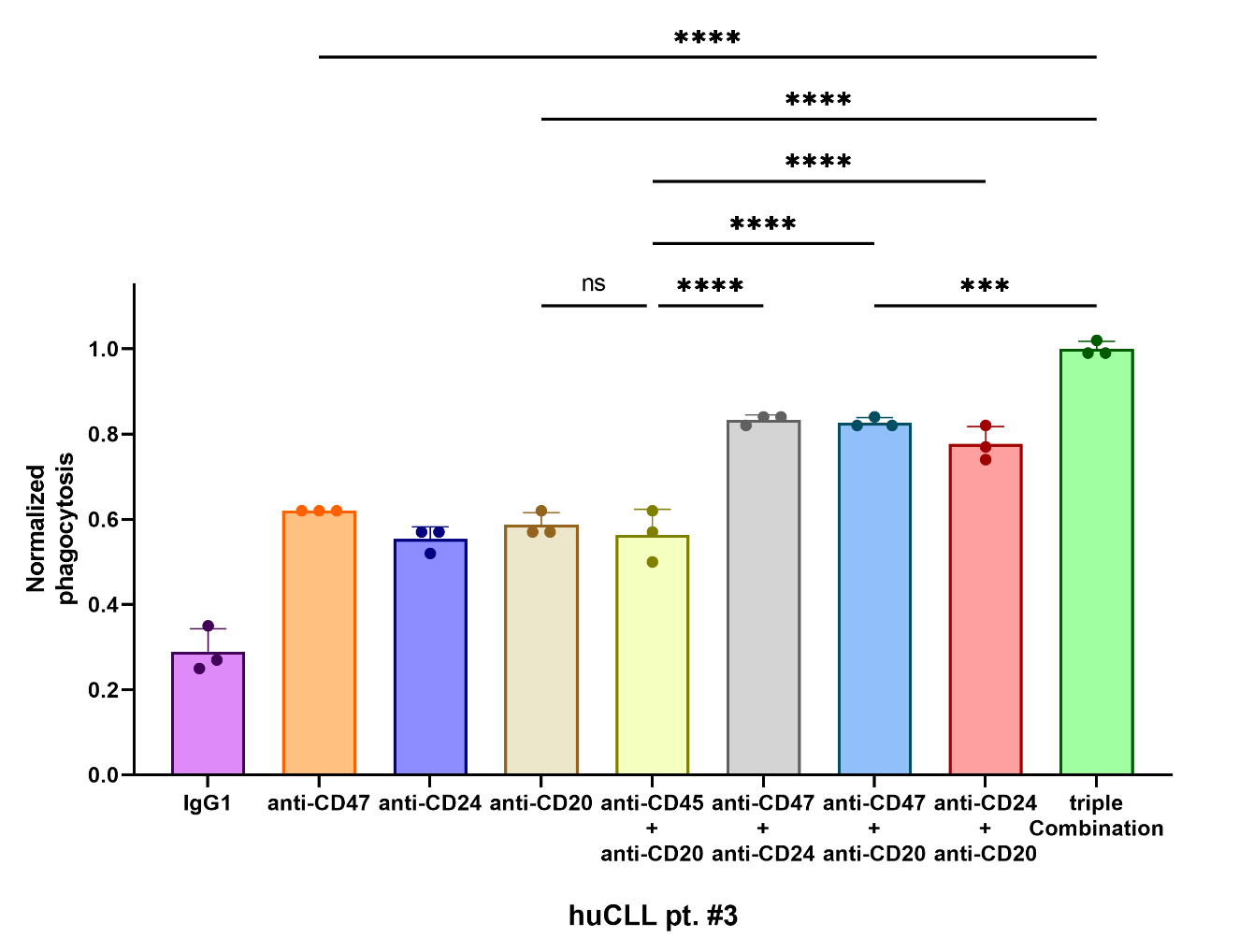


**E**


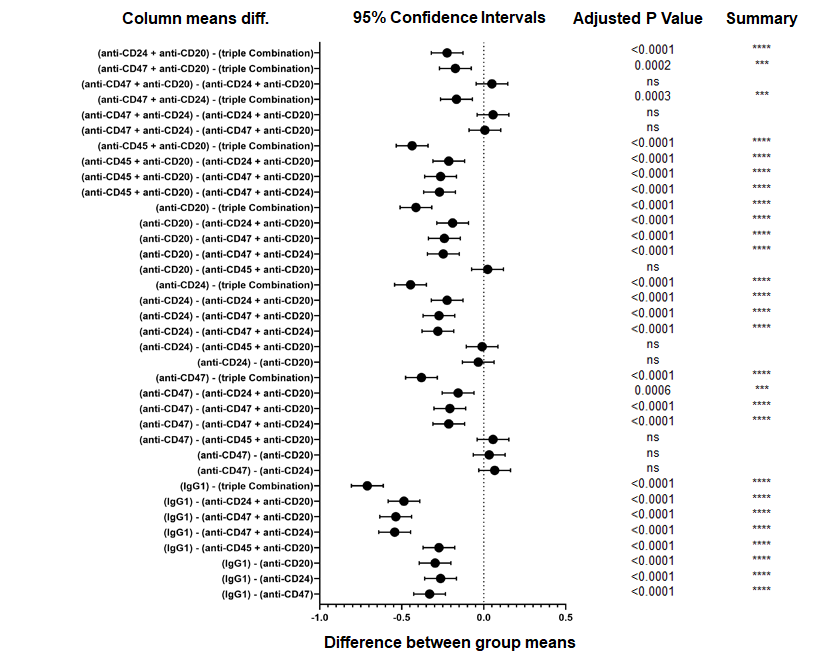


**Supplemental Figure 11**

**Supplemental Figure 11**

**Co-culture analysis of patients-derived samples with “anti-CD45 and anti-CD20 mAbs” condition as confirmation of increase of phagocytosis secondary to DEMs blockade. A-E)** In the setting of patients-derived samples, these experiments with donor macrophages were performed to rule out that previous documented increase of phagocytosis in case of double mAbs combination was simply due to addition of two cumulative Fc-mediated opsonization mechanisms rather than double DEMs blockade or single DEMs blockade associated with Rituximab-mediated opsonization action. Additional co-culture analysis, considering the condition "anti-CD45 + anti-CD20 mAbs", were performed to address this issue. As expected, this condition had similar phagocytic rate in comparison with “anti-CD20 alone” and lower levels of phagocytosis if compared to the other double-mAb conditions. These data suggest that the increase of phagocytosis, when anti-CD47 and/or anti-CD24 were adopted, was due to a different mechanism, like DEMs blockade, rather than combining more antibodies together (one-way ANOVA with multiple comparisons correction; huMCL pt. #1 *F*_(8,18)_ = 936.1, huMCL pt. #2 *F*_(8,18)_ = 1177, huCLL pt. #1 *F*_(8,18)_ = 689.2, huCLL pt. #2 *F*_(8,18)_ = 51.99, huCLL pt. #3 *F*_(8,18)_ = 114.8; technical triplicate, one representative donor; ns: not significant; * p < 0.05, ** p < 0.01, *** p < 0.001, **** p < 0.0001).
